# Efficacy and safety of histone deacetylase inhibitors in peripheral T-cell lymphoma: a systematic review and meta-analysis on prospective clinical trials

**DOI:** 10.3389/fonc.2023.1127112

**Published:** 2023-06-13

**Authors:** Peipei Yang, Yali Tao, Ailin Zhao, Kai Shen, He Li, Jinjin Wang, Hui Zhou, Zhongwang Wang, Mengyao Wang, Ying Qu, Li Zhang, Yuhuan Zheng, Ting Niu

**Affiliations:** Department of Hematology, West China Hospital, Sichuan University, Chengdu, Sichuan, China

**Keywords:** peripheral T-cell lymphoma, subtype, histone deacetylase inhibitor, efficacy, meta-analysis

## Abstract

**Background:**

The overall survival of peripheral T-cell lymphoma (PTCL) is dismal. Histone deacetylase (HDAC) inhibitors have exhibited promising treatment outcomes for PTCL patients. Therefore, this work aims to systematically evaluate the treatment outcome and safety profile of HDAC inhibitor-based treatment for untreated and relapsed/refractory (R/R) PTCL patients.

**Methods:**

The prospective clinical trials of HDAC inhibitors for the treatment of PTCL were searched on the Web of Science, PubMed, Embase, ClinicalTrials.gov, and Cochrane Library database. The pooled overall response rate, complete response (CR) rate, and partial response rate were measured. The risk of adverse events was evaluated. Moreover, the subgroup analysis was utilized to assess the efficacy among different HDAC inhibitors and efficacy in different PTCL subtypes.

**Results:**

For untreated PTCL, 502 patients in seven studies were involved, and the pooled CR rate was 44% (95% *CI*, 39-48%). For R/R PTCL patients, there were 16 studies included, and the CR rate was 14% (95% *CI*, 11-16%). The HDAC inhibitor-based combination therapy exhibited better efficacy when compared with HDAC inhibitor monotherapy for R/R PTCL patients (*P* = 0.02). In addition, the pooled CR rate was 17% (95% *CI*, 13-22%), 10% (95% *CI*, 5-15%), and 10% (95% *CI*, 5-15%) in the romidepsin, belinostat, and chidamide monotherapy subgroups, respectively. In the R/R angioimmunoblastic T-cell lymphoma subgroup, the pooled ORR was 44% (95% *CI*, 35-53%), higher than other subtypes. A total of 18 studies were involved in the safety assessment of treatment-related adverse events. Thrombocytopenia and nausea were the most common hematological and non-hematological adverse events, respectively.

**Conclusion:**

This meta-analysis demonstrated that HDAC inhibitors were effective treatment options for untreated and R/R PTCL patients. The combination of HDAC inhibitor and chemotherapy exhibited superior efficacy to HDAC inhibitor monotherapy in the R/R PTCL setting. Additionally, HDAC inhibitor-based therapy had higher efficacy in angioimmunoblastic T-cell lymphoma patients than that in other subtypes.

## Introduction

Peripheral T-cell lymphoma (PTCL) is a non-Hodgkin lymphoma that originates from post-thymic T-cells and presents with high heterogeneity. As stated by the latest classification of lymphoid neoplasms, PTCLs comprise various subtypes that exhibit different and complex clinicopathologic manifestations (1). Common subtypes include PTCL not otherwise specified (PTCL-NOS), angioimmunoblastic T-cell lymphoma (AITL), and anaplastic lymphoma kinase-negative anaplastic large-cell lymphoma (ALCL, ALK-neg). Most PTCLs exhibit an aggressive clinical course and inferior prognosis. A study that enrolled 166 untreated PTCL patients with a median follow-up of five years indicated that the rate of five-year overall survival (OS) of PTCL patients was 51% (2). In relapsed/refractory (R/R) PTCL patients, the median progression-free survival and median OS were 3.1 months and 5.5 months, respectively, as reported by a retrospective analysis (3). In a prospective study, the median OS of PTCL patients was only 5.8 months after relapse, demonstrating a dismal outcome (4).

The current frontline treatment of PTCL involves combination chemotherapy, typically including CHOP (cyclophosphamide, doxorubicin, vincristine, and prednisone) or CHOP-like regimen. Brentuximab vedotin has also been approved for the treatment of CD30-positive PTCL. Compared to CHOP, adding brentuximab vedotin to the CHP (cyclophosphamide, doxorubicin, prednisone) regimen has shown a meaningful improvement in the 5-year OS rate for CD30-positive PTCL patients, with a rate of 70.1% versus 61.0% (5). However, for subtypes such as PTCL-NOS and AITL, the prognosis remains unsatisfying. Therefore, there is a critical need for advanced therapeutic agents to improve the outcome.

Histone deacetylases (HDACs) are indispensable to controlling protein deacetylation in cells. They play a vital role in the epigenetic modulation of biological processes and regulate protein degradation (6, 7). However, the abnormal activity of HDACs is often related to disease progression (8), leading to increased attention to HDACs as therapeutic targets (9). Preclinical studies have demonstrated that HDAC inhibitors can inhibit tumor cell growth by apoptosis, cell cycle arrest, and cytokine regulation (10, 11). In addition, HDAC inhibitors exhibited antitumor efficacy in solid tumors and hematological malignancies (12, 13). Romidepsin is the first HDAC inhibitor approved for R/R PTCL across all subtypes. Romidepsin primarily inhibits class I HDACs and has a weak effect on class IIb HDACs (14). Another HDAC inhibitor, belinostat, can broadly inhibit all zinc-dependent HDAC enzymes and has been indicated for R/R PTCL (15). Chidamide, which can selectively target the catalytic pocket and inhibit class I and IIb HDACs, also showed promising efficacy in R/R PTCL treatment (16).

Clinical studies have been conducted to evaluate the safety and treatment outcomes of HDAC inhibitor monotherapy or HDAC inhibitor combined with conventional regimens for untreated or R/R PTCL patients in recent years. However, a comprehensive and quantitative meta-analysis of prospective trials evaluating the efficacy and safety of HDAC inhibitors is still lacking. This study aims to bridge the gap by providing a quantitative examination of the efficacy of HDAC inhibitor-based treatment for untreated or R/R PTCL patients, respectively. In the subgroup analysis, we compared the efficacy of monotherapy and combination therapy for R/R PTCL. The treatment outcome of different HDAC inhibitors has also been compared. Additionally, the treatment efficacy in different PTCL subtypes has been analyzed, in order to provide reliable evidence for the optimized application of HDAC inhibitors.

## Method

### Search strategy

This study was reported in obedience to the Preferred Reporting Items for Systematic Reviews and Meta-Analyses statement. In order to identify studies evaluating HDAC inhibitor monotherapy and HDAC inhibitor-based combination therapy in PTCL patients, searches were conducted on various databases including Web of Science, PubMed, Embase, ClinicalTrials.gov, and Cochrane Library. The search strategy employed was related to “lymphoma, t-cell, peripheral” and “histone deacetylase inhibitor”, with detailed strategies outlined in the supplemental material. The retrieval time for the search was November 2022, and no language restrictions were imposed.

### Study selection

Two investigators (PY and YT) screened the search results and determined the eligibility independently. And disagreements were discussed and resolved. The studies which met these features were included (1): prospective clinical trials with large sample size (more than ten patients) (2); adult patients treated with HDAC inhibitors (3); the diagnoses including PTCL; and (4) sufficient data of efficacy and safety assessment. The exclusion criteria were as follows (1): a case report, letter, meeting abstract, or comment (2); studies without raw data on efficacy or safety.

### Data extraction

Two investigators (PY and YT) independently collected the data, and resolved any discrepancies through discussion. For each included study, the following information and data were collected, including (1): basic study information, including the title, registration number, year of publication, first authors’ name, and study design (2); key characteristics, including the number of patients, disease state (untreated PTCL patients or R/R PTCL patients), median age and range, disease stage, treatment details, and PTCL subtypes (3); main outcomes, including overall response rate (ORR), complete response (CR) rate, and partial response (PR) rate (4); treatment-related adverse events (AEs) of all grades, and those of grade 3 or higher. For controlled trials, only the data from the HDAC inhibitor-based treatment arm were extracted.

### Quality evaluation

The quality evaluation for the chosen studies was carried out using the Methodological Index for Nonrandomized Studies (MINORS). This index comprises eight items for non-randomized studies and four additional items for comparative studies (17). The scoring criteria were 0, 1, and 2 for not reported, reported but inadequate, and reported and adequate, respectively. Each included study was scored by two independent investigators (JW and HZ). Any discrepancies were resolved by consensus.

### Statistical analysis

The statistical analysis was conducted by Revman 5.4 and STATA14 (Stata Corporation). The *I-squared* test (*I^2^* test) was applied to the assessment of heterogeneity among the included studies. The *I^2^* values of less than 25%, between 25% and 50%, and higher than 50% were considered as low heterogeneity, moderate heterogeneity, and significant heterogeneity, respectively. Additionally, the Galbraith plot was used to identify potential sources of heterogeneity. For studies with *I^2^* higher than 50%, a random effects model was employed, whereas a fixed effects model was used for those with lower *I^2^* values. To address any heterogeneity, subgroup analyses were performed for monotherapy versus combination therapy, different kinds of HDAC inhibitors, and three PTCL subtypes. Publication bias was assessed using the funnel plot. And the sensitivity analysis was performed to evaluate the robustness of the results.

## Results

### Study selection and characteristics

**Figure 1:**
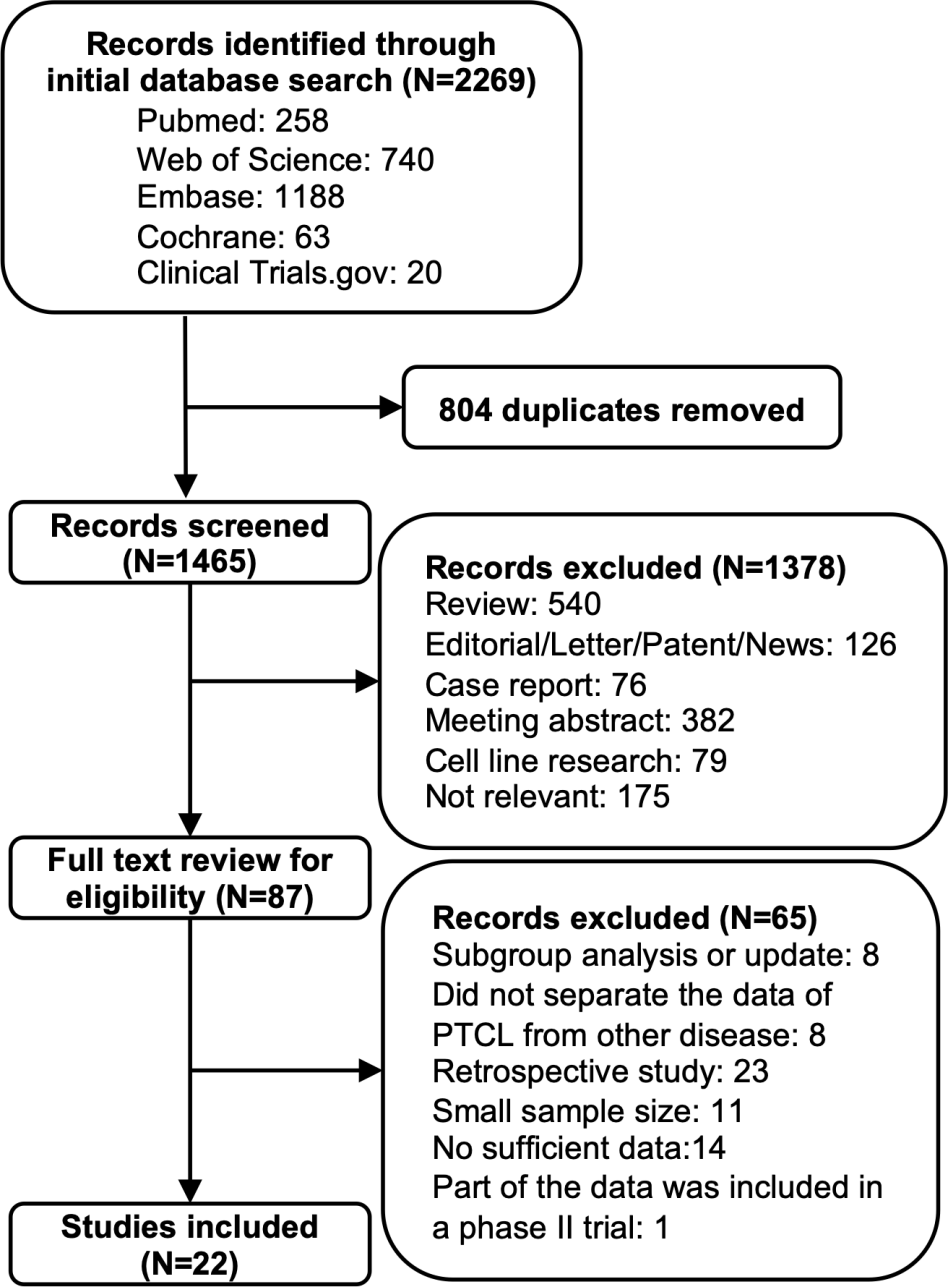
Flow diagram of the process of study search and selection.

We initially identified 1465 studies after the removal of 804 duplications. Then, the screen of the titles and abstracts of these studies was performed, and 87 potentially eligible studies were remained for full-text review and determination. Finally, 22 studies were included. The detailed procedure of study selection is shown in **Figure 1**.

The included studies were conducted from 2011 to 2022, and most of these were phase I or phase II single-arm studies, with only two randomized controlled trials. Among these studies, six enrolled untreated PTCL patients, 15 enrolled R/R PTCL patients, and one enrolled untreated and R/R PTCL patients. The subtypes of PTCL included in these studies were mainly PTCL-NOS, AITL, and ALCL, ALK-neg. In total, 502 untreated patients and 662 R/R patients were involved in our efficacy analysis. For untreated PTCL patients, all of the studies used HDAC inhibitor-based combination therapy, specifically the HDAC inhibitor plus CHOP regimen. Among these studies, three used romidepsin (18–20), one used belinostat (21), and three used chidamide (22–24). For R/R PTCL, nine studies reported HDAC inhibitor monotherapy. Of these, four studies used romidepsin (25–28), two studies used belinostat (29, 30), and three studies used chidamide (12, 31, 32). In addition, seven studies employed combination therapy, including six studies that used romidepsin (20, 33–37) and one study that used panobinostat (38). **Table 1** shows the summarized overall characteristics of the 22 included studies.

**Table 1:** Study characteristics.

Study	Design and Registration number	Patient status	No. of patients	Median age, years (range)	Stage III/IV (%)	HDAC inhibitor	Treatment	T-cell lymphoma subtypes
Dupuis 2015 (18)	Phase I/II, single-arm, NCT01280526	untreated	37	57 (30–77)	95.0	Ro	Ro-CHOP	PTCL-NOS (N=9); AITL (N=15); ALCL, ALK-neg (N=2); Others (N=11)
Bachy 2022 (19)	Phase III, RCT, NCT01796002	untreated	421	65 (25–81)	85.3	Ro	Ro-CHOP	PTCL-NOS (N=59); AITL (N=101); ALCL, ALK-neg (N=21); Others (N=30)
Johnston 2021 (21)	Phase I, single-arm, NCT01839097	untreated	23	63 (35–84)	91.0	Bel	Bel-CHOP	PTCL-NOS (N=9); AITL (N=10); ALCL, ALK-neg (N=1); Others (N=3)
Gui 2021 (22)	Phase I, single-arm, NCT02809573	untreated	30	53 (42–58)	NA	Chidamide	Chidamide-CHOP	PTCL-NOS (N=12); AITL (N=8); ALCL, ALK-neg (N=3); Others (N=7)
Wang 2022 (23)	Phase II, single-arm, NCT03273452	untreated	68	63 (25–83)	82.4	Chidamide	Chidamide-Prednisone+ Etoposide+ Thalidomide	AITL (N=68)
Zhang 2021 (24)	Phase Ib/II, single-arm, NCT02987244	untreated	128	54 (20–70)	80.5	Chidamide	Chidamide-CHOEP	PTCL-NOS (N=49); AITL (N=50); ALCL, ALK-neg (N=21); Others (N=8)
Falchi 2021 (20)	Phase II, single-arm, NCT01998035	untreated R/R	25	63 (42–88)	88.0	Ro	Ro-Azacytidine	PTCL-NOS (N=4); AITL (N=14); Others (N=7)
Piekarz 2011 (25)	Phase II, single-arm, NCT00007345	R/R	47	59 (27–84)	95.7	Ro	Ro	PTCL-NOS (N=27); AITL (N=7); ALCL, ALK-neg (N=2); Others (N=11)
Coiffier 2012 (26)	Phase II, single-arm, NCT00426764	R/R	130	61 (20–83)	70.0	Ro	Ro	PTCL-NOS (N=69); AITL (N=27); ALCL, ALK-neg (N=21); Others (N=13)
Maruyama 2017 (27)	Phase I/II, single-arm, NCT01456039	R/R	50	70 (43–83)	79.0	Ro	Ro	PTCL-NOS (N=20); AITL (N=21); ALCL, ALK-neg (N=3); Others (N=4)
O’Connor 2019 (28)	Phase III, RCT, NCT01482962	R/R	271	63 (19–86)	88.0	Ro	Ro	PTCL-NOS (N=12); AITL (N=6); ALCL, ALK-neg (N=3); Others (N=2)
O’Connor 2015 (29)	Phase II, single-arm, NCT00865969	R/R	120	64 (29–81)	85.0	Bel	Bel	PTCL-NOS (N=77); AITL (N=22); ALCL, ALK-neg (N=13); Others (N=8)
Foss 2015 (30)	Phase II, single-arm, NCT00274651	R/R	53	64 (23–76)	75.0	Bel	Bel	PTCL-NOS (N=13); AITL (N=3); ALCL, ALK-neg (N=3); Others (N=5)
Shi 2015 (31)	Phase II, single-arm, NA	R/R	79	59 (20–77)	82.0	Chidamide	Chidamide	PTCL-NOS (N=27); AITL (N=10); ALCL, ALK-neg (N=11); Others (N=31)
Utsunomiya 2022 (32)	Phase II, single-arm, NCT02955589	R/R	23	72 (60–89)	NA	Chidamide	Chidamide	ATLL (N=23)
Rai 2022 (12)	Phase II, single-arm, NCT02953652	R/R	55	71 (38–87)	NA	Chidamide	Chidamide	PTCL-NOS (N=37); AITL (N=10); ALCL, ALK-neg (N=3); Others (N=5)
Pellegrini 2016 (33)	Phase II, single-arm, NCT01822886	R/R	20	55 (24–77)	95.0	Ro	Ro-Gemcitabine	PTCL-NOS (N=10); AITL (N=9); ALCL, ALK-neg (N=1)
Amengual 2018 (34)	Phase I, single-arm, NCT01947140	R/R	29	54 (23–73)	NA	Ro	Ro-Pralatrexate	PTCL-NOS (N=1); ALCL, ALK-neg (N=3); Others (N=14)
Reiman 2019 (35)	Phase I, single-arm, NCT01846390	R/R	20	65 (48–72)	NA	Ro	Ro-GDP	PTCL-NOS (N=5); AITL (N=2); Others (N=3)
Vu 2020 (36)	Phase I, single-arm, NCT01902225	R/R	24	63 (52–83)	83.0	Ro	Ro-Liposomal Doxorubicin	PTCL-NOS (N=5); AITL (N=4); ALCL, ALK-neg (N=2); Others (N=1)
Mehta-Shah 2021 (37)	Phase Ib/IIa, comparative, NCT01755975/NCT02341014	R/R	76	NA	NA	Ro	Ro-Lenalidomide/Ro-Lenalidomide + Carfilzomib	PTCL-NOS (N=12); AITL (N=8); Others (N=10)
Tan 2015 (38)	Phase II, single-arm, NCT00901147	R/R	25	59 (35–79)	84.0	Panobinostat	Panobinostat-Bortezomib	PTCL-NOS (N=9); AITL (N=8); ALCL, ALK-neg (N=4); Others (N=4)

Ro, romidepsin; Bel, belinostat; CHOP, cyclophosphamide, doxorubicin, vincristine, and prednisone; CHOEP, etoposide with CHOP; R/R, relapsed/refractory; GDP, gemcitabine, dexamethasone and cisplatin; RCT, randomized controlled trial; PTCL-NOS, peripheral T-cell lymphoma not otherwise specified; AITL, angioimmunoblastic T-cell lymphoma; ALCL, ALK-neg, anaplastic lymphoma kinase-negative anaplastic large-cell lymphoma; ATLL, adult T-cell leukemia/lymphoma; NA, not available.

### Quality assessment

All the included studies were evaluated by MINORS, and the detailed scores are shown in **Figure S1**. All of the studies reported the study purpose and successively enrolled patients, and the follow-up period was appropriate. However, most studies did not declare an unbiased endpoint assessment. According to the total scores, seven studies were of high quality (19, 23, 26, 28, 31–33), 11 studies were of moderate quality (12, 18, 21, 22, 24, 27, 29, 30, 35, 36, 38), and four studies were of low quality (20, 25, 34, 37).

### Efficacy in untreated PTCL patients

Seven studies were analyzed to determine the efficacy of HDAC inhibitor-based combination therapy for untreated PTCL patients, and the studies reported ORR, CR rate, and PR rate. In total, the ORR of the included 502 patients was 72% (95% *CI*, 63-82%, random effect model). Of the seven studies, one study used belinostat-based treatment and enrolled 21 patients (21), resulting in an ORR of 86%. Three studies used chidamide-based treatment and totally included 224 patients (22–24), and the pooled ORR was 74% (95% *CI*, 57-92%). Three studies used romidepsin-based treatment and enrolled 257 patients (18–20), resulting in a pooled ORR of 64% (95% *CI*, 58-70%). The analysis revealed a significant difference in ORR among the three HDAC inhibitor-based combination therapies in untreated PTCL patients (*P* = 0.02) (**Figure S2**).

**Figure 2:**
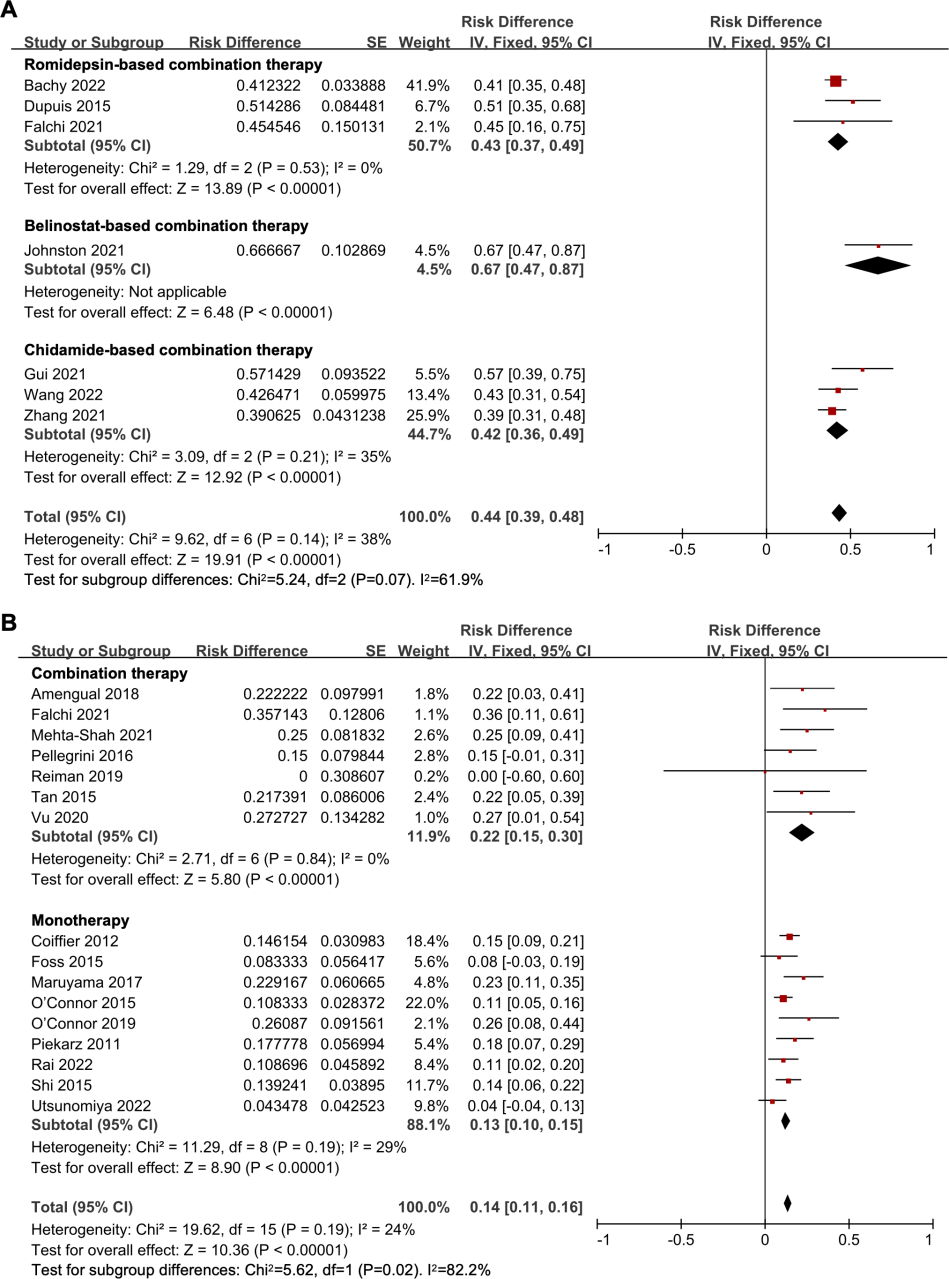
The forest plot of complete response rates in **(A)** untreated peripheral T-cell lymphoma patients and **(B)** relapsed/refractory peripheral T-cell lymphoma patients.

The pooled CR rate was 44% (95% *CI*, 39-48%, fixed effect model). Upon subgroup analysis, the CR rate for the belinostat-based treatment study was 67%. In the romidepsin-based treatment subgroup, the pooled CR was 43% (95% *CI*, 37-49%). For the chidamide-based therapy, the CR rate was 42% (95% *CI*, 36-49%). And the subgroup analysis indicated no statistical difference in CR rate among the different treatment options (*P* = 0.07) (**Figure 2A**).

Moreover, the pooled PR rate was 22% (95% *CI*, 19-26%, fixed effect model). For the subgroup analysis, the PR rate for belinostat-based treatment study was 19%. For romidepsin-based treatment, the estimated PR rate was 21% (95% *CI*, 16-26%). And for chidamide-based treatment group, the estimated PR rate was 24% (95% *CI*, 19-30%), rendering no significant difference in subgroup analysis (*P* = 0.62) (**Figure S3**).

### Efficacy in R/R PTCL patients

The pooled efficacy analysis for R/R PTCL involved 16 studies. The ORR was 37% (95% *CI*, 31-42%, random effect model). In the HDAC inhibitor-based combination therapy subgroup, the ORR was higher at 45% (95% *CI*, 36-54%), whereas the HDAC inhibitor monotherapy subgroup showed a lower ORR at 33% (95% *CI*, 27-38%). The efficacy of HDAC inhibitor-based combination therapy was found superior to HDAC inhibitor monotherapy for R/R PTCL patients (*P* = 0.02) (**Figure S4**). Additionally, in the panobinostat-based combination treatment subgroup, the ORR was 43%. And the ORR of romidepsin-based combination treatment was 46% (95% *CI*, 36-55%), with no statistical difference between the two subgroups (*P* = 0.86) (**Figure S5**). Regarding monotherapy, subgroup analysis indicated that the pooled ORR was 32% (95% *CI*, 27-38%), 26% (95% *CI*, 19-33%), and 33% (95% *CI*, 26-41%) in the romidepsin, belinostat, and chidamide monotherapy subgroups, respectively. And the subgroup analysis revealed no significant difference (*P* = 0.28) (**Figure S6**).

**Figure 3:**
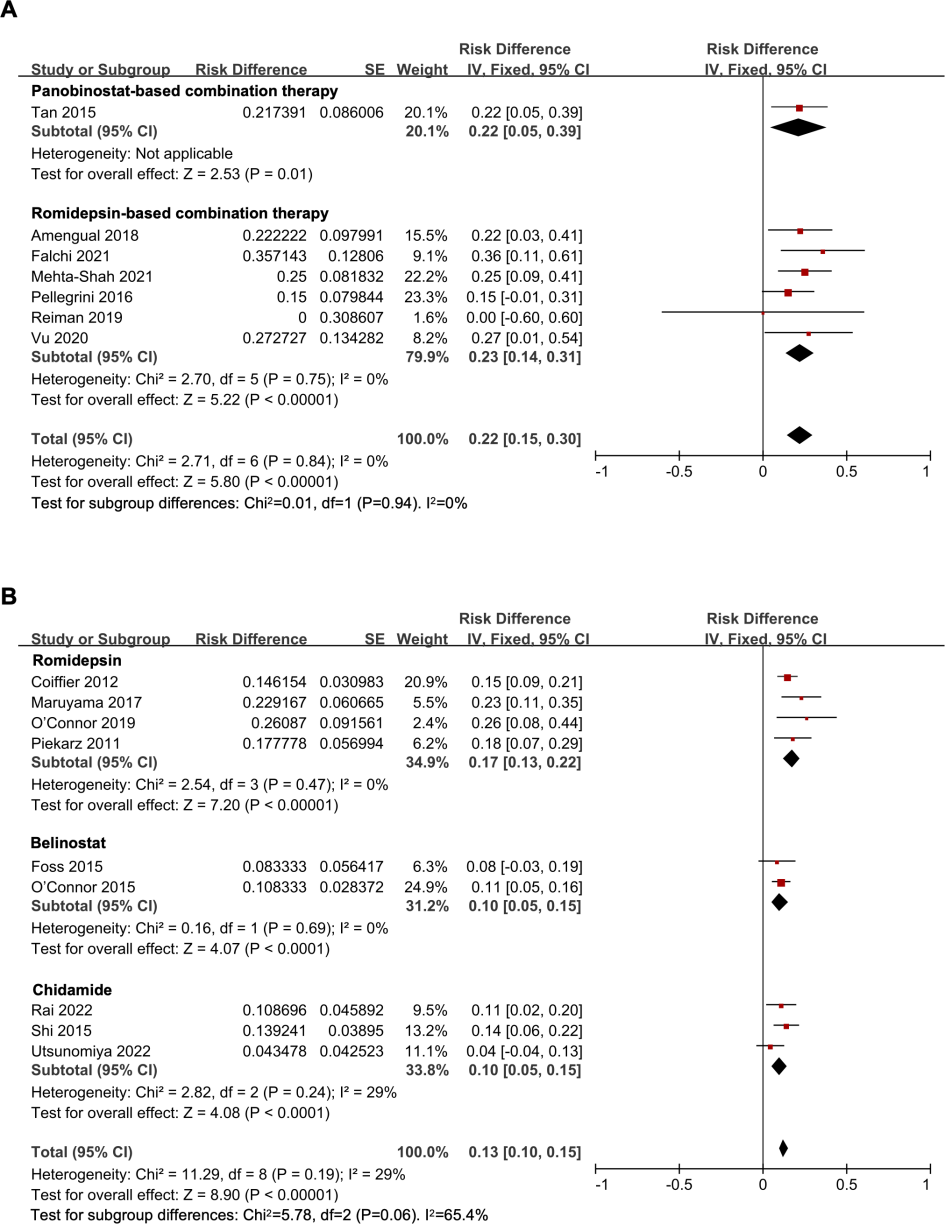
The forest plot of pooled complete response rate of **(A)** histone deacetylase inhibitor-based combination therapy and **(B)** histone deacetylase inhibitor monotherapy therapy in relapsed/refractory peripheral T-cell lymphoma patients.

The overall pooled CR rate was 14% (95% *CI*, 11-16%, fixed effect model). In the combination therapy group, the CR rate was 22% (95% *CI*, 15-30%). And in the HDAC inhibitor monotherapy subgroup, the CR rate was 13% (95% *CI*, 10-15%), which was significantly lower than that of the combination therapy (*P* = 0.02) (**Figure 2B**). The CR rate of panobinostat-based combination therapy was 22%, and the CR rate of romidepsin-based combination therapy was 23% (95% *CI*, 14-31%), with no statistical difference (*P* = 0.94) (**Figure 3A**). In addition, the pooled CR rate for monotherapy was 17% (95% *CI*, 13-22%), 10% (95% *CI*, 5-15%), and 10% (95% *CI*, 5-15%) in the romidepsin, belinostat, and chidamide monotherapy subgroups, respectively. No statistical discrepancy was observed in the CR rate among the three types of HDAC inhibitors (*P* = 0.06) (**Figure 3B**).

The overall pooled PR rate of the included 16 trials was 17% (95% *CI*, 14-20%, fixed effect model). In the combination therapy, the PR rate was 24% (95% *CI*, 16-31%), while the HDAC inhibitor monotherapy subgroup, the PR rate was 16% (95% *CI*, 13-19%), with no statistical difference (*P* = 0.06) (**Figure S7**). The PR rate of the panobinostat-based combination therapy and the romidepsin-based combination therapy was 22% and 24% (95% *CI*, 16-33%), respectively. This subgroup analysis revealed no significant difference (*P* = 0.80) (**Figure S8**). In addition, subgroup analysis of monotherapy indicated that the pooled PR rate were 14% (95% *CI*, 10-18%), 15% (95% *CI*, 9-21%), and 20% (95% *CI*, 13-26%) in the romidepsin, belinostat, and chidamide monotherapy, respectively, without a statistical difference (*P* = 0.35) (**Figure S9**).

### Efficacy in PTCL subtypes

**Figure 4:**
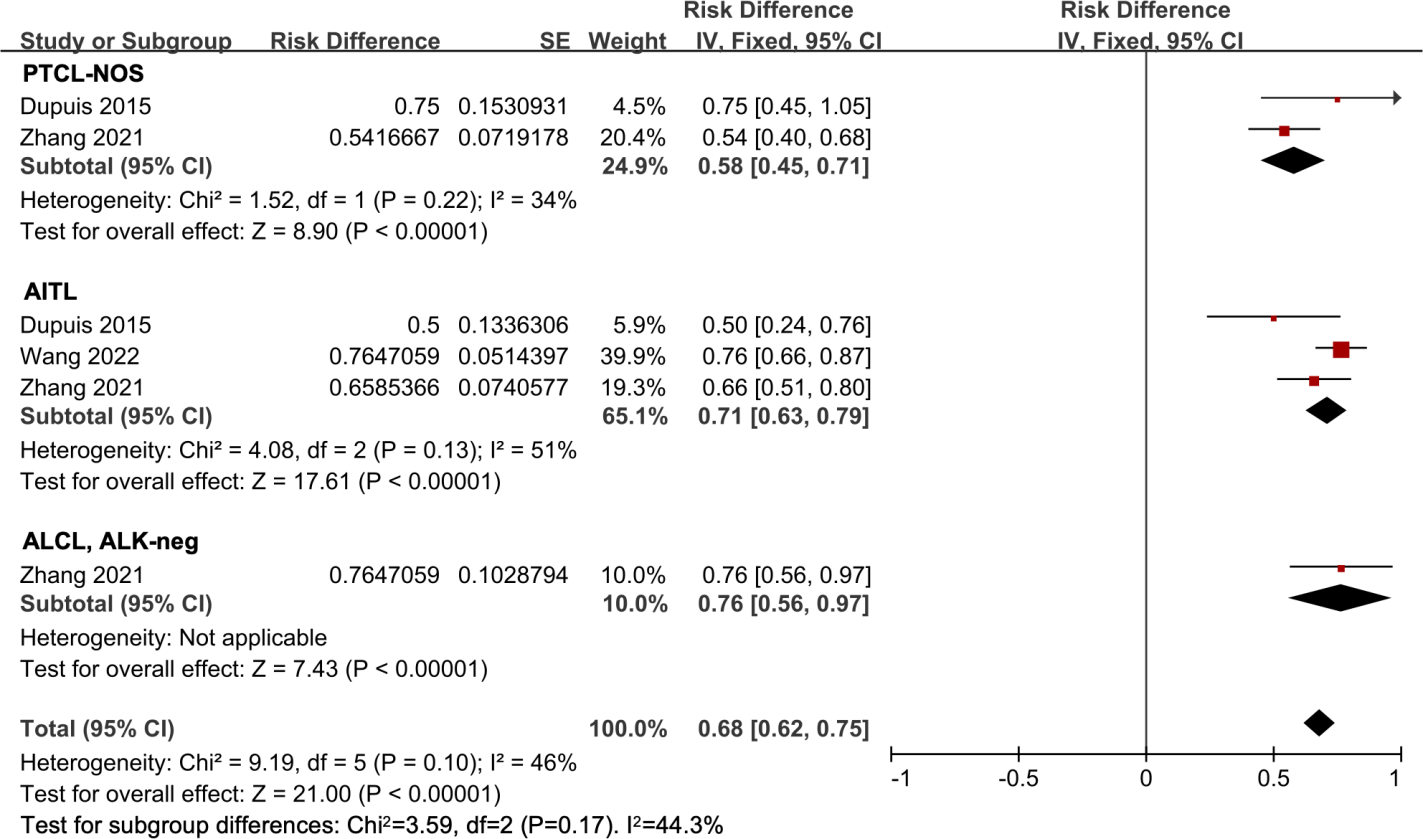
The forest plot of pooled overall response rate in different subtypes in untreated peripheral T-cell lymphoma patients. PTCL-NOS, peripheral T-cell lymphoma not otherwise specified; AITL, angioimmunoblastic T-cell lymphoma; ALCL, ALK-neg, anaplastic lymphoma kinase-negative anaplastic large-cell lymphoma.

There were three studies involved in the subtype analysis in untreated PTCL patients (18, 23, 24). The ORR of all 196 patients was 68% (95% CI, 62-75%, fixed effect model). Of these, the pooled ORR in the PTCL-NOS, AITL, and ALCL, ALK-neg subgroups were 58% (95% *CI*, 45-71%), 71% (95% *CI*, 63-79%), and 76% (95% *CI*, 56-97%), respectively. However, no significant difference was observed (*P* = 0.17) (**Figure 4**).

**Figure 5:**
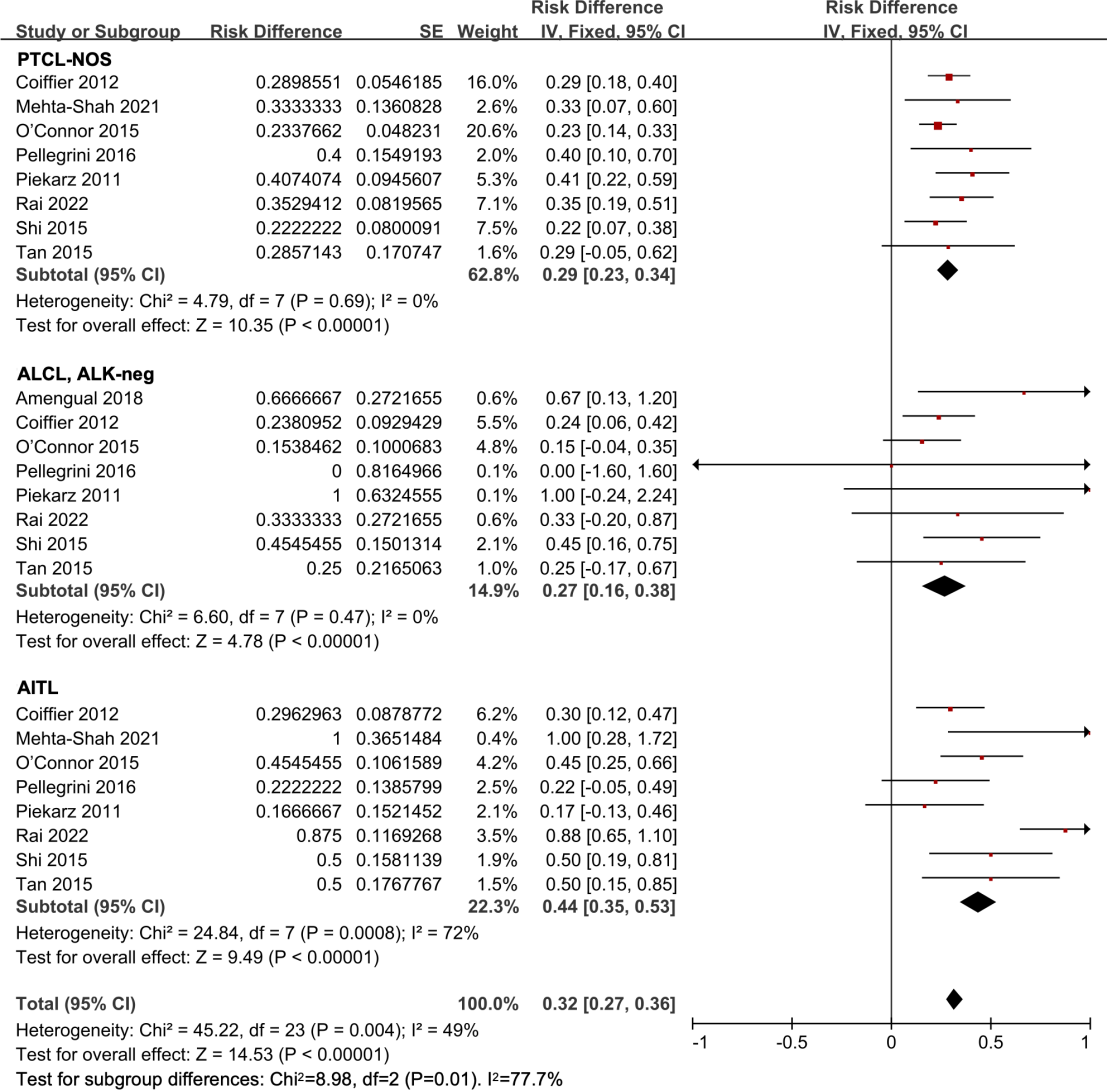
The forest plot of pooled overall response rate in different subtypes in relapsed/refractory peripheral T-cell lymphoma patients. PTCL-NOS, peripheral T-cell lymphoma not otherwise specified; AITL, angioimmunoblastic T-cell lymphoma; ALCL, ALK-neg, anaplastic lymphoma kinase-negative anaplastic large-cell lymphoma.

Eight studies were included in the histological subtype analysis in R/R PTCL patients (12, 25, 26, 29, 31, 33, 34, 37). The ORR of the all 418 patients was 32% (95% *CI*, 27-36%, fixed effect model). In the subgroup analysis, the pooled ORR was 29% (95% *CI*, 23-34%) in the PTCL-NOS patients. The pooled ORR in the ALCL, ALK-negative patients was 27% (95% *CI*, 16-38%). And in the AITL subgroup, the pooled ORR was 44% (95% *CI*, 35-53%), indicating a more efficient response compared to other subgroups (*P* = 0.01) (**Figure 5**).

### Safety evaluation

Eighteen studies were included for safety assessment (12, 18–33, 38). The AEs were graded in compliance with the National Cancer Institute Common Toxicity Criteria for AEs (CTCAE) (Version 3.0, 4.0, or 4.03). Thrombocytopenia was the most common, with a pooled incidence rate of 54% (95% *CI*, 32-77%) for all grades. Other common hematological AEs included neutropenia and leukopenia, and the incidence rates were 50% (95% *CI*, 32-67%) and 46% (95% *CI*, 24-69%), respectively. The most common grade 3 or higher hematological AE was neutropenia. Nausea was the most frequent non-hematological AE for all grades, with an incidence rate of 53% (95% *CI*, 36-70%). Moreover, nausea, fever/pyrexia, decreased appetite, and hypokalemia were common grade 3 or higher non-hematological AEs. The pooled incidence rate and the number of included studies in every AE are listed in **Table 2**.

**Table 2:** Pooled incidence of treatment-related adverse events.

Adverse events	Treatment		All grades			Grades 3		
			Included study (n)	Pooled rate [95%CI]	*P* value	Included study (n)	Pooled rate [95%CI]	*P* value
Anemia	Combination therapy		9	55% [39%, 70%]	**0.001**	9	22% [9%, 36%]	**0.03**
	Monotherapy		8	24% [13%, 34%]		8	6% [3%, 9%]	
	Overall		17	40% [24%, 57%]		17	15% [9%, 21%]	
Thrombocytopenia	Combination therapy		8	62% [45%, 80%]	0.43	8	43% [24%, 62%]	0.05
	Monotherapy		8	46% [9%, 83%]		8	21% [10%, 32%]	
	Overall		16	54% [32%, 77%]		16	32% [21%, 43%]	
Neutropenia	Combination therapy		8	59% [42%, 77%]	0.12	9	54% [40%, 68%]	**0.001**
	Monotherapy		6	37% [15%, 59%]		6	23% [11%, 35%]	
	Overall		14	50% [32%, 67%]		15	41% [27%, 56%]	
Leukopenia	Combination therapy		4	57% [21%, 92%]	0.43	4	49% [18%, 81%]	0.07
	Monotherapy		6	39% [15%, 63%]		6	19% [9%, 29%]	
	Overall		10	46% [24%, 69%]		10	32% [15%, 48%]	
Lymphopenia	Combination therapy		3	25% [14%, 36%]	0.42	3	21% [6%, 36%]	0.38
	Monotherapy		3	45% [-1%, 91%]		3	38% [3%, 72%]	
	Overall		6	36% [13%, 60%]		6	30% [13%, 46%]	
Fatigue	Combination therapy		6	38% [20%, 56%]	0.11	4	1% [0%, 3%]	**0.02**
	Monotherapy		7	22% [12%, 31%]		2	10% [3%, 17%]	
	Overall		13	29% [20%, 37%]		6	2% [0%, 3%]	
Fever/Pyrexia	Combination therapy		7	24% [16%, 32%]	0.66	3	7% [4%, 11%]	0.19
	Monotherapy		8	22% [11%, 32%]		2	4% [1%, 7%]	
	Overall		15	23% [15%, 30%]		5	5% [3%, 8%]	
Nausea	Combination therapy		4	78% [70%, 85%]	**0.0003**	3	11% [5%, 18%]	**0.006**
	Monotherapy		8	41% [24%, 59%]		2	2% [0%, 4%]	
	Overall		12	53% [36%, 70%]		5	5% [1%, 9%]	
Vomiting	Combination therapy		7	41% [19%, 64%]	0.18	5	7% [4%, 9%]	**0.004**
	Monotherapy		6	23% [10%, 37%]		3	2% [0%, 4%]	
	Overall		13	33% [21%, 44%]		8	3% [2%, 5%]	
Diarrhea	Combination therapy		5	31% [10%, 53%]	0.48	2	2% [-1%, 5%]	0.84
	Monotherapy		8	23% [15%, 31%]		3	2% [0%, 3%]	
	Overall		13	26% [17%, 34%]		5	2% [0%, 3%]	
Constipation	Combination therapy		5	21% [6%, 35%]	0.87	2	2% [-1%, 4%]	—
	Monotherapy		5	19% [10%, 28%]		0	—	
	Overall		10	20% [11%, 28%]		2	2% [-1%, 4%]	
Decrease appetite	Combination therapy		5	26% [19%, 34%]	0.57	3	6% [3%, 8%]	0.29
	Monotherapy		5	32% [13%, 51%]		4	4% [1%, 6%]	
	Overall		10	29% [20%, 38%]		7	5% [3%, 6%]	
Hypokalemia	Combination therapy		4	26% [14%, 38%]	**0.004**	3	8% [3%, 13%]	**0.03**
	Monotherapy		3	7% [3%, 11%]		1	2% [-1%, 4%]	
	Overall		7	18% [10%, 27%]		4	6% [1%, 10%]	

The bold values indicated for significant differences (P<0.05).

The incidence rate of AEs of HDAC inhibitor monotherapy and HDAC inhibitor-based combination therapy of PTCL were compared. Subgroup analysis of AEs of all grades showed that combination therapy exhibited higher rates of anemia (55% vs. 24%, *P* = 0.001), nausea (78% vs. 41%, *P* = 0.0003), and hypokalemia (26% vs. 7%, *P* = 0.004), compared to monotherapy. Moreover, the subgroup analysis of grade 3 or higher AEs suggested that combination therapy showed higher occurrences of anemia (22% vs. 6%, *P* = 0.03), neutropenia (54% vs. 23%, *P* = 0.001), nausea (11% vs. 2%, *P* = 0.006), vomiting (7% vs. 2%, *P* = 0.004), and hypokalemia (8% vs. 2%, *P* = 0.03), but the lower occurrence of fatigue (1% vs. 10%, *P* = 0.02), when compared to HDAC inhibitor monotherapy.

### Publication bias, heterogeneity analysis, and sensitivity test

**Figure 6:**
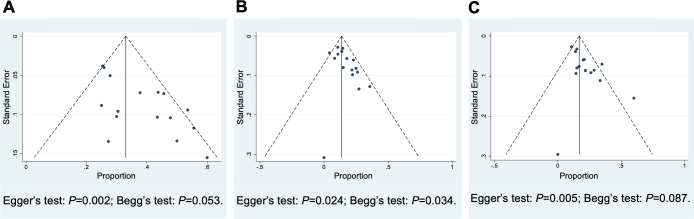
Funnel Plots of the studies included in relapsed/refractory peripheral T-cell lymphoma to estimate the publication bias. **(A)** Overall response rate, **(B)** complete response rate, **(C)** partial response rate.

Funnel plots were utilized to estimate the publication bias of the studies of R/R PTCL. Asymmetry was found in the results of ORR, CR rate, and PR rate, suggesting evidence of publication bias (**Figure 6**). The *I^2^* test and Galbraith plots indicated that heterogeneity existed in the outcome of ORR of untreated PTCL, ORR and PR rate of R/R PTCL. And low risks of heterogeneity were found in the results of CR and PR rate of untreated PTCL, and CR rate of R/R PTCL (**Figures S10-S15**). The sensitivity analysis was conducted by omitting four low-quality studies (20, 25, 34, 37). For untreated PTCL patients, the pooled ORR was 73% (95% *CI*, 63-83%, random effect model) (**Figure S16**). And for R/R PTCL, the pooled ORR was 31% (95% *CI*, 27-35%, fixed effect model). Although combination therapy exhibited a higher ORR than monotherapy, no statistical discrepancy was found in the sensitivity analysis (*P* = 0.19) (**Figure S17**).

## Discussion

To address the poor outcome of PTCL, several agents have been evaluated in clinical trials, including monoclonal antibodies, hypomethylating agents, PI3K/Akt/mTOR pathway inhibitors, and HDAC inhibitors (39). And HDAC inhibitors have been extensively studied for the treatment of PTCL. Thus, in this paper, we conducted a systematic review of prospective clinical trials and a meta-analysis to evaluate the efficacy and safety profile of HDAC inhibitors for PTCL.

We analyzed the treatment efficacy in untreated PTCL patients by including seven studies, resulting in a pooled ORR of 72% and a pooled CR rate of 44%, which demonstrates high therapeutic efficacy. There was a significant difference in ORR among three HDAC inhibitor-based combination therapies (*P* = 0.02). The belinostat-based combination therapy demonstrated the best ORR. The chidamide-based combination therapy performed well, while the romidepsin-based combination therapy showed an inferior outcome to other HDAC inhibitors. These three HDAC inhibitors have different structures and various target specificities, as well as distinct pharmacokinetic and pharmacodynamic properties, which may lead to different outcomes (31). It’s noteworthy that only one study was included in the belinostat group, with a small sample size. Therefore, it is necessary to verify the efficacy of belinostat-based therapy in larger samples of untreated PTCL patients in the future. Five studies used HDAC inhibitor plus CHOP or CHOEP (CHOP plus etoposide) regimen. The CHOP or CHOP-like regimen is typically the frontline therapy for newly diagnosed PTCL patients. Our analysis included a controlled trial, which indicated that the ORR of CHOP therapy was 60%, and the ORR of the romidepsin plus CHOP therapy was 63%, with no significant difference observed (19). However, the duration of response in CR or PR patients was prolonged with the treatment of romidepsin plus CHOP. In a double-blind, randomized trial that enrolled previously untreated PTCL patients, the ORR was 72% with the CHOP therapy (40). In addition, a real-world study demonstrated that the ORR was 75% with CHOEP treatment and 65% with CHOP treatment (41). Moreover, in a retrospective analysis, untreated PTCL patients achieved an ORR of 82.6%, 76.1%, and 75.0% when treated with CHOP, CHOEP, and CHOEP alternating with a gemcitabine-based regimen, respectively (42). Although HDAC inhibitor-based combination therapies resulted in a high ORR in untreated PTCL, no additional benefits have been found with the treatment of HDAC inhibitor plus chemotherapy when indirectly compared with the CHOP or CHOP-like regimen. Thus, more controlled clinical trials are needed to identify promising therapies for untreated PTCL patients.

The clinical outcome is generally dismal in R/R PTCL patients. In our study, 16 studies enrolling 665 R/R PTCL patients were included, and most patients were at stage III or IV. Our findings suggested that the HDAC inhibitors have favorable efficacy in R/R PTCL patients, with an estimated ORR of 37%, a CR rate of 14%, and a PR rate of 17%. Furthermore, subgroup analysis indicated that HDAC inhibitor-based combination therapy showed significantly higher ORR and CR rate (45% and 23%, respectively) than HDAC inhibitor monotherapy. A meta-analysis was conducted to evaluate the treatment outcome and tolerability of novel drugs versus chemotherapy in R/R T-cell lymphoma. The ORR of single novel agents in R/R PTCL was 36%, which was lower than that of chemotherapy and the combination of chemotherapy and novel agents (43), which is partly consistent with our findings. No statistical differences were found in the subgroup analysis of the efficacy of different HDAC inhibitors, while in the monotherapy, romidepsin and chidamide exhibited slightly higher efficacy than belinostat. Chidamide, which was developed in China, showed promising therapeutic capacity. A meta-analysis involving prospective and retrospective studies of PTCL patients treated with romidepsin showed a CR rate of 20%. Controversially, the study found no statistical difference comparing romidepsin monotherapy with romidepsin-based combination therapy (44). The discrepancy may be attributed to the difference that our study included four kinds of HDAC inhibitor-related studies and conducted a more comprehensive analysis. Another study summarized the efficacy of belinostat for PTCL, and the result was consistent with our study (45).

The efficacy of HDAC inhibitors varies across different subtypes of PTCL. In untreated PTCL patients, the AITL and ALCL, ALK-neg subgroups showed higher ORR compared to the PTCL-NOS subgroup. In the R/R PTCL setting, HDAC inhibitors exhibited favorable efficacy in the AITL subgroup, while the outcome was still suboptimal in the PTCL-NOS subgroup. These results suggested that HDAC inhibitors were more effective in patients with AITL. Several genetic mutations have been found in AITL, including *TET2, DNMT3A*, and *RHOA (* 46, 47). The mutations in epigenetic regulation genes may relate to the promising activity of epigenetic drugs (48). Identifying the genomic and molecular information, and exploring the relationship between therapeutic activity and disease subtypes can further enhance the precise management of the disease.

The analysis of treatment-related AEs indicated that hematological AEs frequently occurred, including thrombocytopenia, neutropenia, and leukopenia. Among these, thrombocytopenia was the most common AE, and a preclinical study reported that HDAC inhibitors decreased the release of platelets from megakaryocytes (49). Furthermore, the analysis of grade 3 or higher AEs indicated that hematological AEs occurred more frequently than non-hematological AEs. Most AEs were reversible, and supportive care was typically provided. Dose modification can be utilized to manage these AEs. Compared to HDAC inhibitor monotherapy, the HDAC inhibitor-based combination therapy showed a higher incidence of hematological AEs, including anemia and neutropenia. In a clinical trial, the romidepsin plus CHOP regimen resulted in severe AEs of hematological, gastrointestinal, and nutritional origin. Additionally, the addition of romidepsin led to frequent dose reduction, dose interruption, and low dose intensity of the CHOP regimen (19). Therefore, managing AEs and adjusting drug dosage are crucial when using combination therapy. Although some non-hematological AEs, such as nausea, vomiting, fatigue, and diarrhea, occurred frequently, they were primarily graded 1-2, with a low incidence of grade 3 or higher. The results are consistent with another meta-analysis that included romidepsin-related studies (44).

However, there are still inevitable limitations in this work. First, most of the included studies were single-arm trials and lacked comparability. Second, the studies used different versions of CTCAE, including version 3.0, 4.0, and 4.03. Additionally, four of the included studies had a MINORS score of nine, indicating low quality, which may affect the reliability of our findings.

In conclusion, our systematic review and meta-analysis demonstrates that HDAC inhibitors are effective treatment options for both untreated and R/R PTCL patients. The combination of HDAC inhibitor and chemotherapy exhibited superior efficacy to HDAC inhibitor monotherapy in the R/R PTCL setting. Moreover, HDAC inhibitor-based therapy was more effective in AITL patients than in other subtypes. Further research is needed, particularly randomized controlled trials, to robustly evaluate the treatment outcomes of HDAC inhibitors compared with other therapies. The potential for HDAC inhibitors combined with novel agents should also be explored.

## Data availability statement

The original contributions presented in the study are included in the article/**Supplementary Material**. Further inquiries can be directed to the corresponding author.

## Author contributions

TN conceived the ideas of the manuscript and supervised the writing. PY and YT designed the study, searched the literature, extracted and analyzed the data, and drafted the initial manuscript. AZ designed the study and revised the manuscript. KS, HL, JW, and HZ assessed the quality of included studies and analyzed the data. ZW, MW, and YQ checked the collected data and revised the manuscript for intellectual content. LZ and YZ revised the manuscript and supervised the writing. All authors contributed to the article and approved the submitted version.
