# Supplementary material for: Efficacy and safety of histone deacetylase inhibitors in peripheral T-cell lymphoma: a systematic review and meta-analysis on prospective clinical trials
Source: Front Oncol. 2023 Jun 13;13:1127112. doi: 10.3389/fonc.2023.1127112 (PMC10293743; doi:10.3389/fonc.2023.1127112)
Supplement: Supplementary file 1 [file DataSheet_1.pdf]

Supplemental material

Method

Search strategy

Web of Science: 740 results

1 TS=(lymphoma, t-cell, peripheral)
2 TS=(Peripheral AND (T-cell OR T cell) AND lymphoma)
3 TS=(PTCL)
4 #3 OR #2 OR #1
5 TS=(histone deacetylase inhibitor)
TS=(((((Romidepsin) OR (Belinostat)) OR (Chidamide)) OR (Vorinostat)) OR
6 (panobinostat))
7 #6 OR #5
8 #7 AND #4

Pubmed: 258 results

(((PTCL) OR (Peripheral AND (T-cell OR T cell) AND lymphoma)) OR (lymphoma, t-cell, peripheral[MeSH Terms])) AND (((histone deacetylase inhibitor[MeSH Terms]) OR (histone deacetylase inhibitor[Title/Abstract])) OR (((((Romidepsin) OR (Belinostat)) OR (Chidamide)) OR (Vorinostat)) OR (panobinostat)))

Embase: 1188 results

1 peripheral T cell lymphoma/
2 (Peripheral and (T-cell or T cell) and lymphoma).mp.
3 PTCL).mp.
4 1 or 2 or 3
5 histone deacetylase inhibitor/
6 (Romidepsin or Belinostat or Chidamide or Vorinostat or panobinostat).mp.
7 5 or 6
8 4 and 7

Cochrane Central Register of Controlled Trials Library database: 63 results

1 MeSH descriptor: [Lymphoma, T-Cell, Peripheral] explode all trees
2 Peripheral AND (T-cell OR T cell) AND lymphoma
3 PTCL
4 #1 OR #2 OR #3
5 MeSH descriptor: [histone deacetylase inhibitors] explode all trees
6 HDAC inhibitor
(((((Romidepsin) OR (Belinostat)) OR (Chidamide)) OR (Vorinostat)) OR
7 (panobinostat))
8 #5 OR #6 OR #7
8 #4 AND #8

Figure S1.

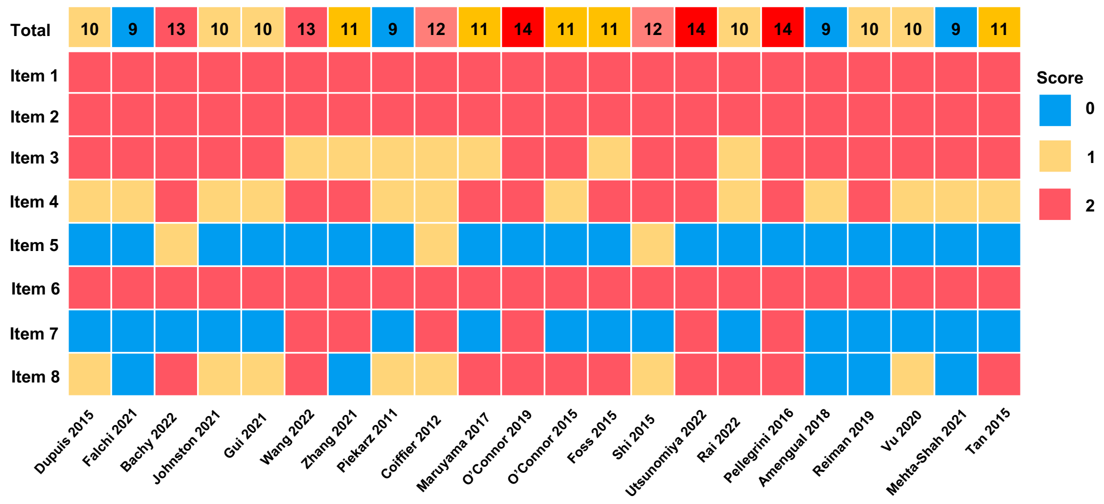

Figure S1. MINORS evaluation for the included studies. 0 for not reported, 1 for reported but inadequate, 2 for reported and adequate. Item 1, A clearly stated aim; Item 2, Inclusion of consecutive patients; Item 3, Prospective collection of data; Item 4, Endpoints appropriate to the aim of the study; Item 5, Unbiased assessment of the study endpoint; Item 6, Follow-up period appropriate to the aim of the study; Item 7, Loss to follow up less than 5%; Item 8, Prospective calculation of the study size.

Figure S2.

Study or Subgroup	Risk Difference	SE	Weight	Risk Difference IV, Random, 95% CI
**Belinostat-based combination therapy**				
Johnston 2021	0.857143	0.07636	13.2%	0.86 [0.71, 1.01]
**Subtotal (95% CI)**			**13.2%**	**0.86 [0.71, 1.01]**
Heterogeneity: Not applicable				
Test for overall effect: Z = 11.23 (P < 0.00001)				
**Chidamide-based combination therapy**				
Gui 2021	0.892857	0.058451	15.3%	0.89 [0.78, 1.01]
Wang 2022	0.764706	0.05144	16.2%	0.76 [0.66, 0.87]
Zhang 2021	0.585938	0.043537	17.1%	0.59 [0.50, 0.67]
**Subtotal (95% CI)**			**48.6%**	**0.74 [0.57, 0.92]**
Heterogeneity: Tau² = 0.02; Chi² = 18.97, df = 2 (P < 0.0001); I² = 89%				
Test for overall effect: Z = 8.23 (P < 0.00001)				
**Romidepsin-based combination therapy**				
Bachy 2022	0.630332	0.033232	18.2%	0.63 [0.57, 0.70]
Dupuis 2015	0.685714	0.078469	13.0%	0.69 [0.53, 0.84]
Falchi 2021	0.636364	0.145041	7.0%	0.64 [0.35, 0.92]
**Subtotal (95% CI)**			**38.2%**	**0.64 [0.58, 0.70]**
Heterogeneity: Tau² = 0.00; Chi² = 0.42, df = 2 (P = 0.81); I² = 0%				
Test for overall effect: Z = 21.33 (P < 0.00001)				
**Total (95% CI)**			**100.0%**	**0.72 [0.63, 0.82]**
Heterogeneity: Tau² = 0.01; Chi² = 28.01, df = 6 (P < 0.0001); I² = 79%				
Test for overall effect: Z = 15.14 (P < 0.00001)				
Test for subgroup differences: Chi²=7.76, df=2 (P=0.02). I²=74.2%				

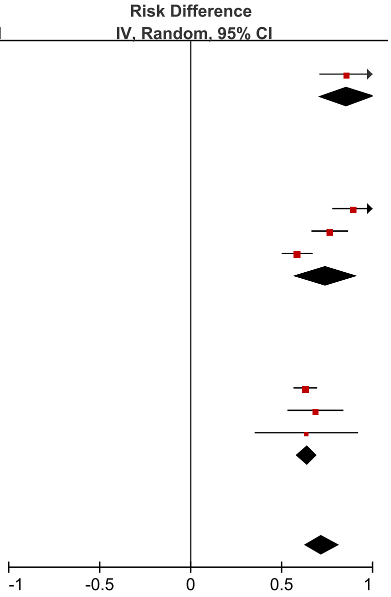

Figure S2. The forest plot of pooled overall response rate in untreated peripheral T-cell lymphoma patients.

Figure S3.

Study or Subgroup	Risk Difference	SE	Weight	Risk Difference IV, Fixed, 95% CI
**Belinostat-based combination therapy**				
Johnston 2021	0.190476	0.085689	4.6%	0.19 [0.02, 0.36]
**Subtotal (95% CI)**			**4.6%**	**0.19 [0.02, 0.36]**
Heterogeneity: Not applicable				
Test for overall effect: Z = 2.22 (P = 0.03)				
**Chidamide-based combination therapy**				
Gui 2021	0.321429	0.088259	4.4%	0.32 [0.15, 0.49]
Wang 2022	0.338235	0.057373	10.3%	0.34 [0.23, 0.45]
Zhang 2021	0.195313	0.035041	27.7%	0.20 [0.13, 0.26]
**Subtotal (95% CI)**			**42.4%**	**0.24 [0.19, 0.30]**
Heterogeneity: Chi² = 5.40, df = 2 (P = 0.07); I² = 63%				
Test for overall effect: Z = 8.58 (P < 0.00001)				
**Romidepsin-based combination therapy**				
Bachy 2022	0.21801	0.028425	42.1%	0.22 [0.16, 0.27]
Dupuis 2015	0.171429	0.063705	8.4%	0.17 [0.05, 0.30]
Falchi 2021	0.181818	0.116291	2.5%	0.18 [-0.05, 0.41]
**Subtotal (95% CI)**			**53.0%**	**0.21 [0.16, 0.26]**
Heterogeneity: Chi² = 0.50, df = 2 (P = 0.78); I² = 0%				
Test for overall effect: Z = 8.25 (P < 0.00001)				
**Total (95% CI)**			**100.0%**	**0.22 [0.19, 0.26]**
Heterogeneity: Chi² = 6.86, df = 6 (P = 0.33); I² = 13%				
Test for overall effect: Z = 12.07 (P < 0.00001)				
Test for subgroup differences: Chi²=0.96, df=2 (P=0.62). I²=0%				

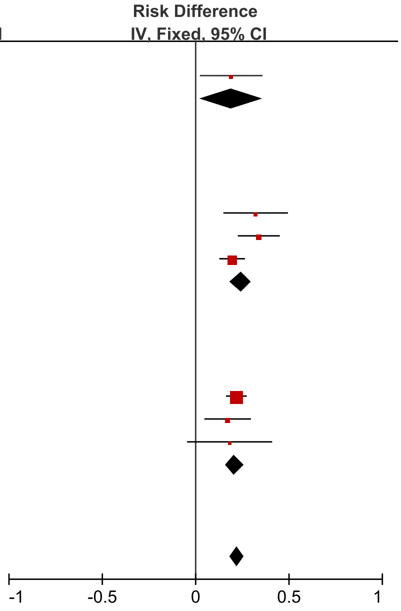

Figure S3. The forest plot of pooled partial response rate in untreated peripheral T-cell lymphoma patients.

Figure S4.

Study or Subgroup	Risk Difference	SE	Weight	Risk Difference IV, Random, 95% CI
**Combination therapy**				
Amengual 2018	0.555556	0.117121	4.2%	0.56 [0.33, 0.79]
Falchi 2021	0.5	0.133631	3.5%	0.50 [0.24, 0.76]
Mehta-Shah 2021	0.535714	0.09425	5.6%	0.54 [0.35, 0.72]
Pellegrini 2016	0.3	0.10247	5.0%	0.30 [0.10, 0.50]
Reiman 2019	0.6	0.154919	2.8%	0.60 [0.30, 0.90]
Tan 2015	0.434783	0.103367	5.0%	0.43 [0.23, 0.64]
Vu 2020	0.272727	0.134282	3.4%	0.27 [0.01, 0.54]
**Subtotal (95% CI)**			**29.5%**	**0.45 [0.36, 0.54]**
Heterogeneity: Tau² = 0.00; Chi² = 6.62, df = 6 (P = 0.36); I² = 9%				
Test for overall effect: Z = 9.82 (P < 0.00001)				
**Monotherapy**				
Coiffier 2012	0.253846	0.038171	11.2%	0.25 [0.18, 0.33]
Foss 2015	0.25	0.088388	6.0%	0.25 [0.08, 0.42]
Maruyama 2017	0.4375	0.071603	7.5%	0.44 [0.30, 0.58]
O'Connor 2015	0.258333	0.039958	11.0%	0.26 [0.18, 0.34]
O'Connor 2019	0.478261	0.104159	4.9%	0.48 [0.27, 0.68]
Piekarz 2011	0.377778	0.072274	7.4%	0.38 [0.24, 0.52]
Rai 2022	0.456522	0.073442	7.3%	0.46 [0.31, 0.60]
Shi 2015	0.278481	0.050432	9.8%	0.28 [0.18, 0.38]
Utsunomiya 2022	0.304348	0.095944	5.5%	0.30 [0.12, 0.49]
**Subtotal (95% CI)**			**70.5%**	**0.33 [0.27, 0.38]**
Heterogeneity: Tau² = 0.00; Chi² = 15.28, df = 8 (P = 0.05); I² = 48%				
Test for overall effect: Z = 11.11 (P < 0.00001)				
**Total (95% CI)**			**100.0%**	**0.37 [0.31, 0.42]**
Heterogeneity: Tau² = 0.01; Chi² = 31.24, df = 15 (P = 0.008); I² = 52%				
Test for overall effect: Z = 12.76 (P < 0.00001)				
Test for subgroup differences: Chi²=5.35, df=1 (P=0.02). I²=81.3%				

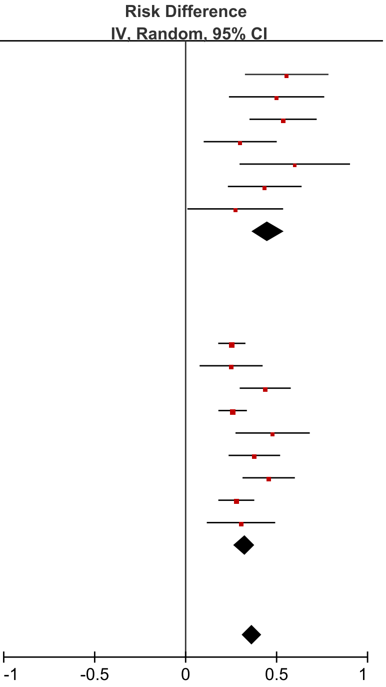

Figure S4. The forest plot of pooled overall response rate in relapsed/refractory peripheral T-cell lymphoma patients.

Figure S5.

Study or Subgroup	Risk Difference	SE	Weight	Risk Difference IV, Fixed, 95% CI
**Panobinostat-based combination therapy**				
Tan 2015	0.434783	0.103367	17.7%	0.43 [0.23, 0.64]
**Subtotal (95% CI)**			**17.7%**	**0.43 [0.23, 0.64]**
Heterogeneity: Not applicable				
Test for overall effect: Z = 4.21 (P < 0.0001)				
**Romidepsin-based combination therapy**				
Amengual 2018	0.555556	0.117121	13.8%	0.56 [0.33, 0.79]
Falchi 2021	0.5	0.133631	10.6%	0.50 [0.24, 0.76]
Mehta-Shah 2021	0.535714	0.09425	21.3%	0.54 [0.35, 0.72]
Pellegrini 2016	0.3	0.10247	18.1%	0.30 [0.10, 0.50]
Reiman 2019	0.6	0.154919	7.9%	0.60 [0.30, 0.90]
Vu 2020	0.272727	0.134282	10.5%	0.27 [0.01, 0.54]
**Subtotal (95% CI)**			**82.3%**	**0.46 [0.36, 0.55]**
Heterogeneity: Chi² = 6.59, df = 5 (P = 0.25); I² = 24%				
Test for overall effect: Z = 9.48 (P < 0.00001)				
**Total (95% CI)**			**100.0%**	**0.45 [0.37, 0.54]**
Heterogeneity: Chi² = 6.62, df = 6 (P = 0.36); I² = 9%				
Test for overall effect: Z = 10.37 (P < 0.00001)				
Test for subgroup differences: Chi²=0.03, df=1 (P=0.86). I²=0%				

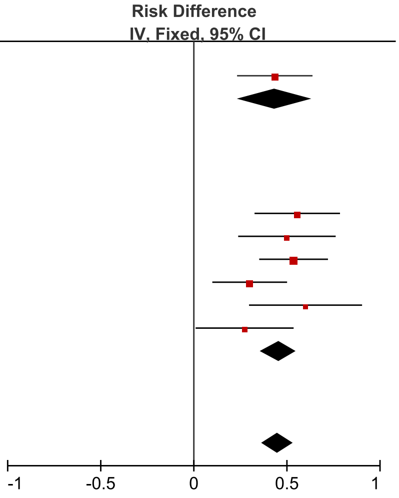

Figure S5. The forest plot of pooled overall response rate of histone deacetylase inhibitor-based combination therapy in relapsed/refractory peripheral T-cell lymphoma patients.

Figure S6.

Study or Subgroup	Risk Difference	SE	Weight	Risk Difference IV, Fixed, 95% CI
**Romidepsin**				
Coiffier 2012	0.253846	0.038171	26.3%	0.25 [0.18, 0.33]
Maruyama 2017	0.4375	0.071603	7.5%	0.44 [0.30, 0.58]
O'Connor 2019	0.478261	0.104159	3.5%	0.48 [0.27, 0.68]
Piekarz 2011	0.377778	0.072274	7.3%	0.38 [0.24, 0.52]
**Subtotal (95% CI)**			**44.7%**	**0.32 [0.27, 0.38]**
Heterogeneity: $Chi^2 = 8.64$, df = 3 (P = 0.03); $I^2 = 65\%$				
Test for overall effect: Z = 11.01 (P < 0.00001)				
**Belinostat**				
Foss 2015	0.25	0.088388	4.9%	0.25 [0.08, 0.42]
O'Connor 2015	0.258333	0.039958	24.0%	0.26 [0.18, 0.34]
**Subtotal (95% CI)**			**28.9%**	**0.26 [0.19, 0.33]**
Heterogeneity: $Chi^2 = 0.01$, df = 1 (P = 0.93); $I^2 = 0\%$				
Test for overall effect: Z = 7.06 (P < 0.00001)				
**Chidamide**				
Rai 2022	0.456522	0.073442	7.1%	0.46 [0.31, 0.60]
Shi 2015	0.278481	0.050432	15.1%	0.28 [0.18, 0.38]
Utsunomiya 2022	0.304348	0.095944	4.2%	0.30 [0.12, 0.49]
**Subtotal (95% CI)**			**26.4%**	**0.33 [0.26, 0.41]**
Heterogeneity: $Chi^2 = 4.08$, df = 2 (P = 0.13); $I^2 = 51\%$				
Test for overall effect: Z = 8.67 (P < 0.00001)				
**Total (95% CI)**			**100.0%**	**0.31 [0.27, 0.34]**
Heterogeneity: $Chi^2 = 15.28$, df = 8 (P = 0.05); $I^2 = 48\%$				
Test for overall effect: Z = 15.61 (P < 0.00001)				
Test for subgroup differences: $Chi^2=2.56$, df=2 (P=0.28). $I^2=21.8\%$				

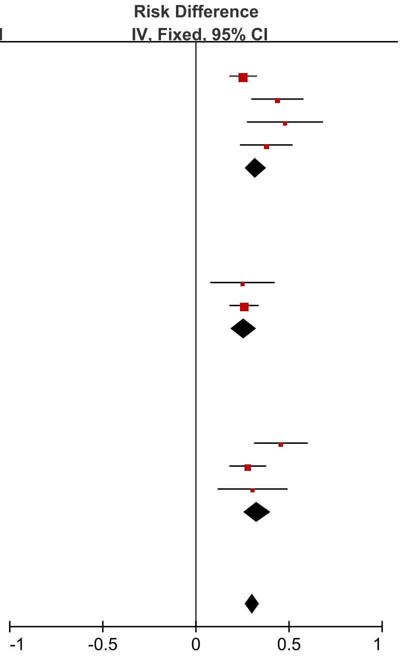

Figure S6. The forest plot of pooled overall response rate of histone deacetylase inhibitor monotherapy in relapsed/refractory peripheral T-cell lymphoma patients.

Figure S7.

Study or Subgroup	Risk Difference	SE	Weight	Risk Difference IV, Fixed, 95% CI
**Combination therapy**				
Amengual 2018	0.333333	0.111111	1.7%	0.33 [0.12, 0.55]
Falchi 2021	0.142857	0.093522	2.4%	0.14 [-0.04, 0.33]
Mehta-Shah 2021	0.285714	0.085374	2.8%	0.29 [0.12, 0.45]
Pellegrini 2016	0.15	0.079844	3.2%	0.15 [-0.01, 0.31]
Reiman 2019	0.6	0.154919	0.9%	0.60 [0.30, 0.90]
Tan 2015	0.217391	0.086006	2.8%	0.22 [0.05, 0.39]
Vu 2020	0	0.294884	0.2%	0.00 [-0.58, 0.58]
**Subtotal (95% CI)**			**14.0%**	**0.24 [0.16, 0.31]**
Heterogeneity: Chi² = 9.47, df = 6 (P = 0.15); I² = 37%				
Test for overall effect: Z = 6.16 (P < 0.00001)				
**Monotherapy**				
Coiffier 2012	0.107692	0.027188	28.0%	0.11 [0.05, 0.16]
Foss 2015	0.166667	0.076073	3.6%	0.17 [0.02, 0.32]
Maruyama 2017	0.208333	0.058618	6.0%	0.21 [0.09, 0.32]
O’Connor 2015	0.15	0.032596	19.5%	0.15 [0.09, 0.21]
O’Connor 2019	0.217391	0.086006	2.8%	0.22 [0.05, 0.39]
Piekarz 2011	0.2	0.059629	5.8%	0.20 [0.08, 0.32]
Rai 2022	0.347826	0.070224	4.2%	0.35 [0.21, 0.49]
Shi 2015	0.139241	0.03895	13.6%	0.14 [0.06, 0.22]
Utsunomiya 2022	0.26087	0.091561	2.5%	0.26 [0.08, 0.44]
**Subtotal (95% CI)**			**86.0%**	**0.16 [0.13, 0.19]**
Heterogeneity: Chi² = 14.01, df = 8 (P = 0.08); I² = 43%				
Test for overall effect: Z = 10.17 (P < 0.00001)				
**Total (95% CI)**			**100.0%**	**0.17 [0.14, 0.20]**
Heterogeneity: Chi² = 27.11, df = 15 (P = 0.03); I² = 45%				
Test for overall effect: Z = 11.73 (P < 0.00001)				
Test for subgroup differences: Chi²=3.64, df=1 (P=0.06). I²=72.5%				

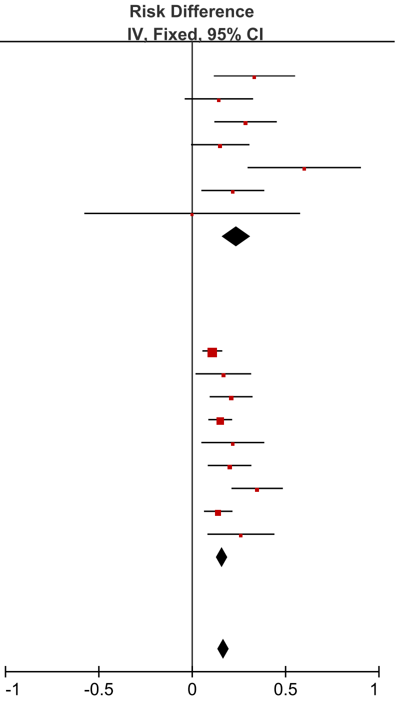

Figure S7. The forest plot of pooled partial response rate in relapsed/refractory peripheral T-cell lymphoma patients.

Figure S8.

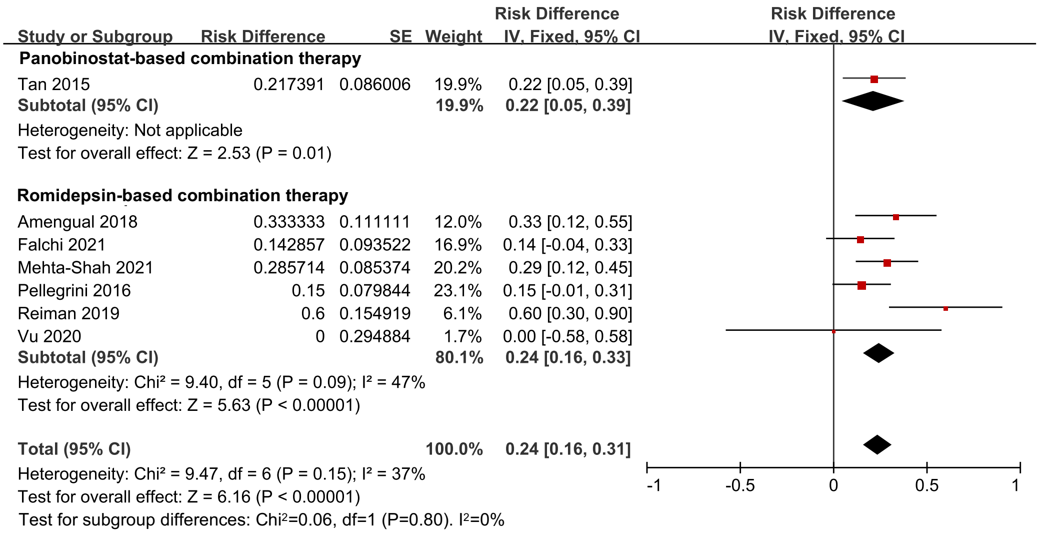

Figure S8. The forest plot of pooled partial response rate of histone deacetylase inhibitor-based combination therapy in relapsed/refractory peripheral T-cell lymphoma patients.

Figure S9.

Study or Subgroup	Risk Difference	SE	Weight	Risk Difference IV, Fixed, 95% CI
**Romidepsin**				
Coiffier 2012	0.107692	0.027188	32.6%	0.11 [0.05, 0.16]
Maruyama 2017	0.208333	0.058618	7.0%	0.21 [0.09, 0.32]
O'Connor 2019	0.217391	0.086006	3.3%	0.22 [0.05, 0.39]
Piekarz 2011	0.2	0.059629	6.8%	0.20 [0.08, 0.32]
**Subtotal (95% CI)**			**49.6%**	**0.14 [0.10, 0.18]**
Heterogeneity: $Chi^2 = 4.59$, df = 3 (P = 0.20); $I^2 = 35\%$				
Test for overall effect: Z = 6.43 (P < 0.00001)				
**Belinostat**				
Foss 2015	0.166667	0.076073	4.2%	0.17 [0.02, 0.32]
O'Connor 2015	0.15	0.032596	22.6%	0.15 [0.09, 0.21]
**Subtotal (95% CI)**			**26.8%**	**0.15 [0.09, 0.21]**
Heterogeneity: $Chi^2 = 0.04$, df = 1 (P = 0.84); $I^2 = 0\%$				
Test for overall effect: Z = 5.09 (P < 0.00001)				
**Chidamide**				
Rai 2022	0.347826	0.070224	4.9%	0.35 [0.21, 0.49]
Shi 2015	0.139241	0.03895	15.9%	0.14 [0.06, 0.22]
Utsunomiya 2022	0.26087	0.091561	2.9%	0.26 [0.08, 0.44]
**Subtotal (95% CI)**			**23.6%**	**0.20 [0.13, 0.26]**
Heterogeneity: $Chi^2 = 7.30$, df = 2 (P = 0.03); $I^2 = 73\%$				
Test for overall effect: Z = 6.18 (P < 0.00001)				
**Total (95% CI)**			**100.0%**	**0.16 [0.13, 0.19]**
Heterogeneity: $Chi^2 = 14.01$, df = 8 (P = 0.08); $I^2 = 43\%$				
Test for overall effect: Z = 10.17 (P < 0.00001)				
Test for subgroup differences: $Chi^2=2.08$, df=2 (P=0.35). $I^2=3.9\%$				

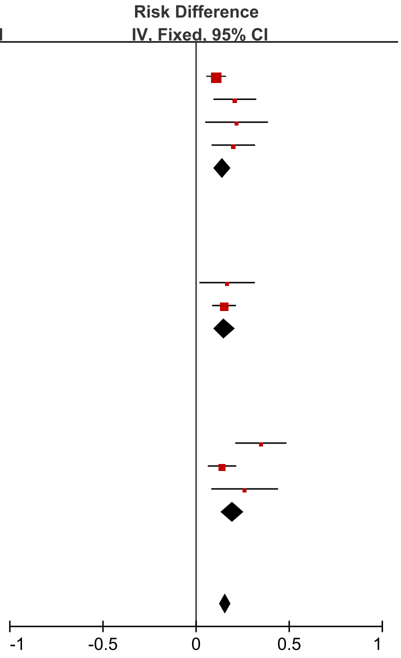

Figure S9. The forest plot of pooled partial response rate of histone deacetylase inhibitor monotherapy in relapsed/refractory peripheral T-cell lymphoma patients.

Figure S10.

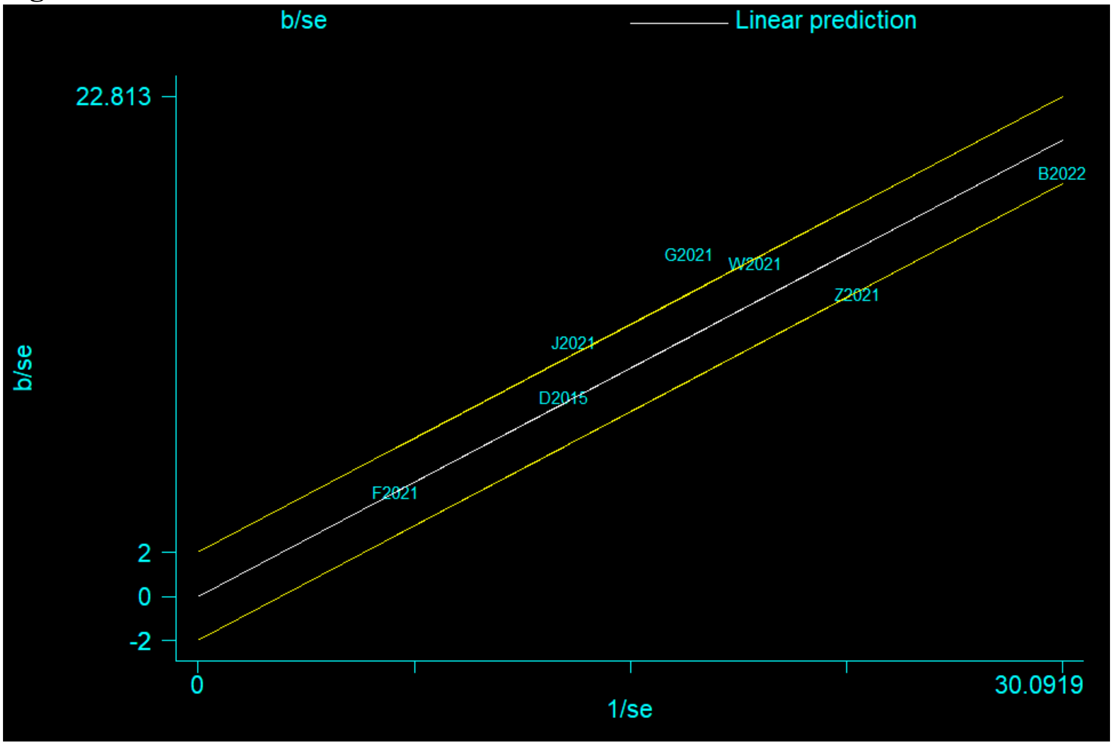

Figure S10. The Galbraith plot of overall response rate of untreated peripheral T-cell lymphoma.

Figure S11.

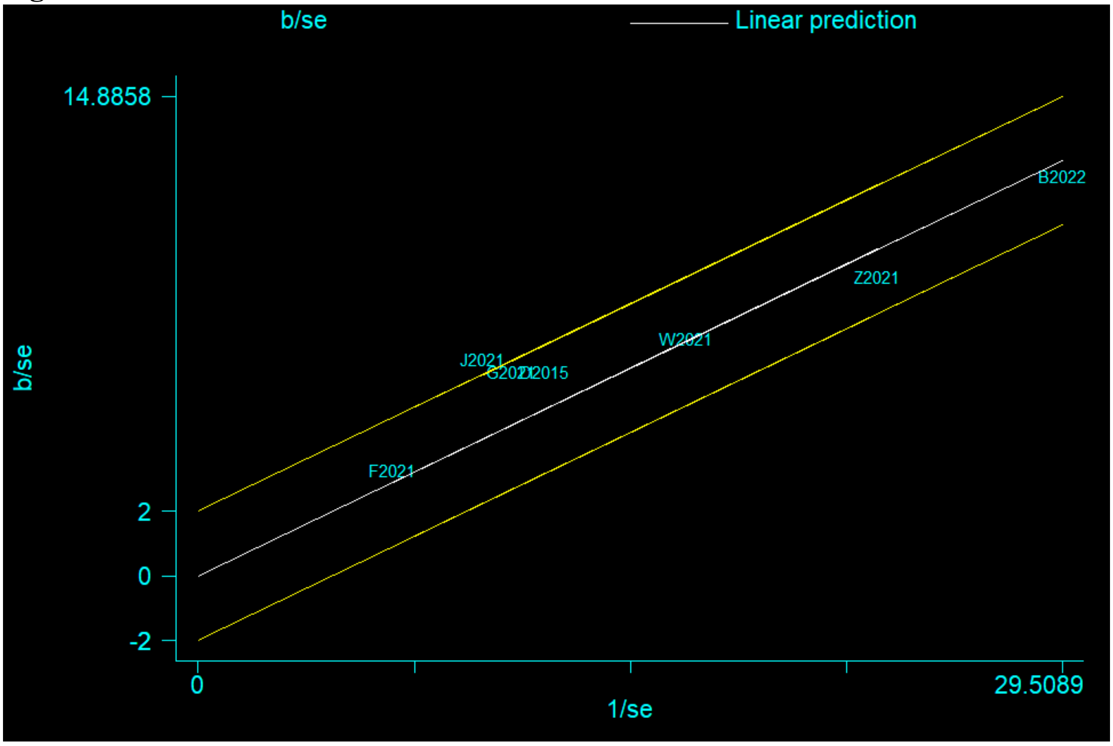

Figure S11. The Galbraith plot of complete response rate of untreated peripheral T-cell lymphoma.

Figure S12.

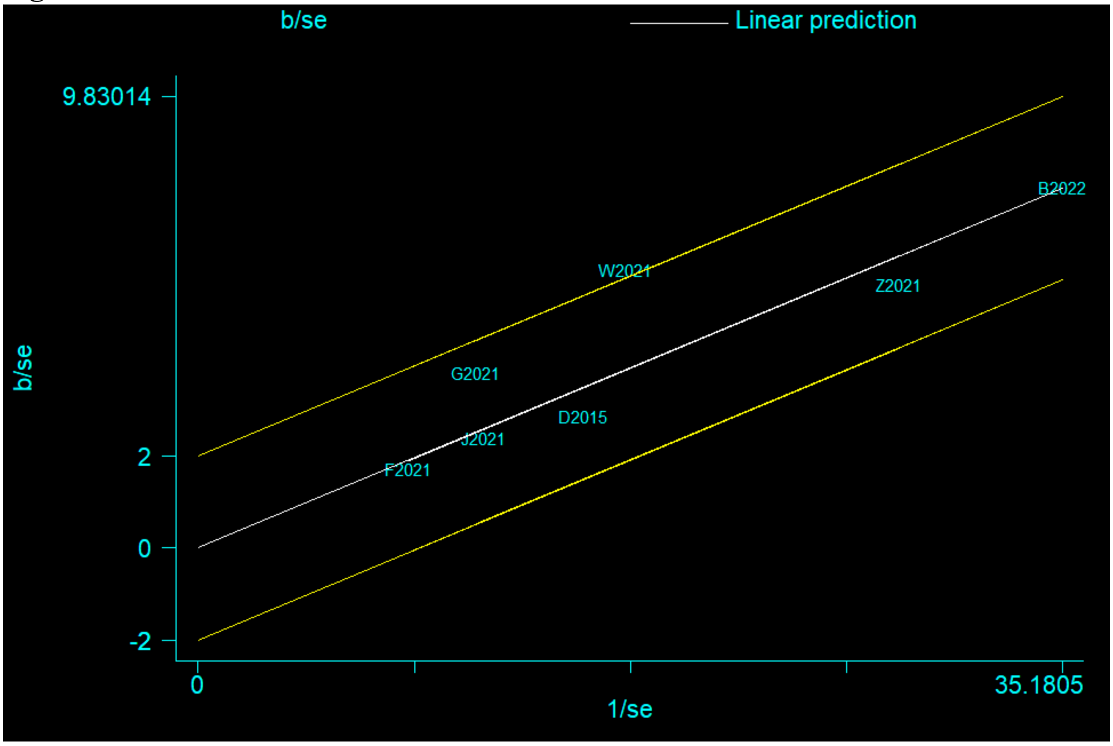

Figure S12. The Galbraith plot of partial response rate of untreated peripheral T-cell lymphoma.

Figure S13.

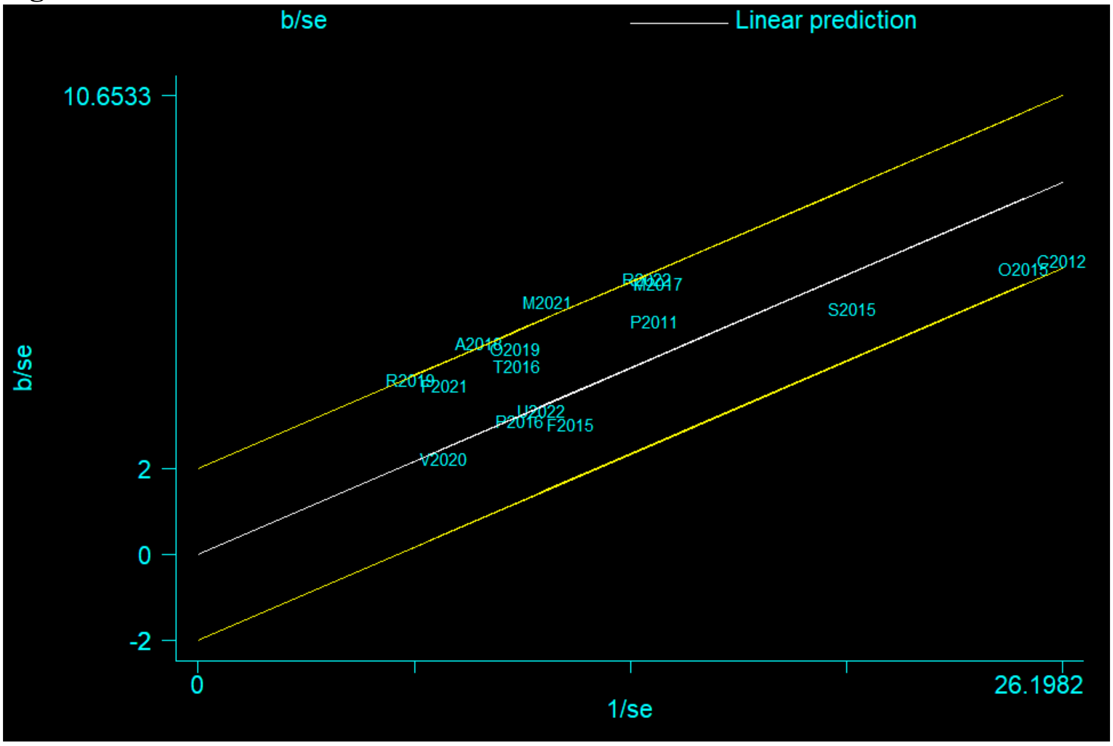

Figure S13. The Galbraith plot of overall response rate of relapsed/refractory peripheral T-cell lymphoma.

Figure S14.

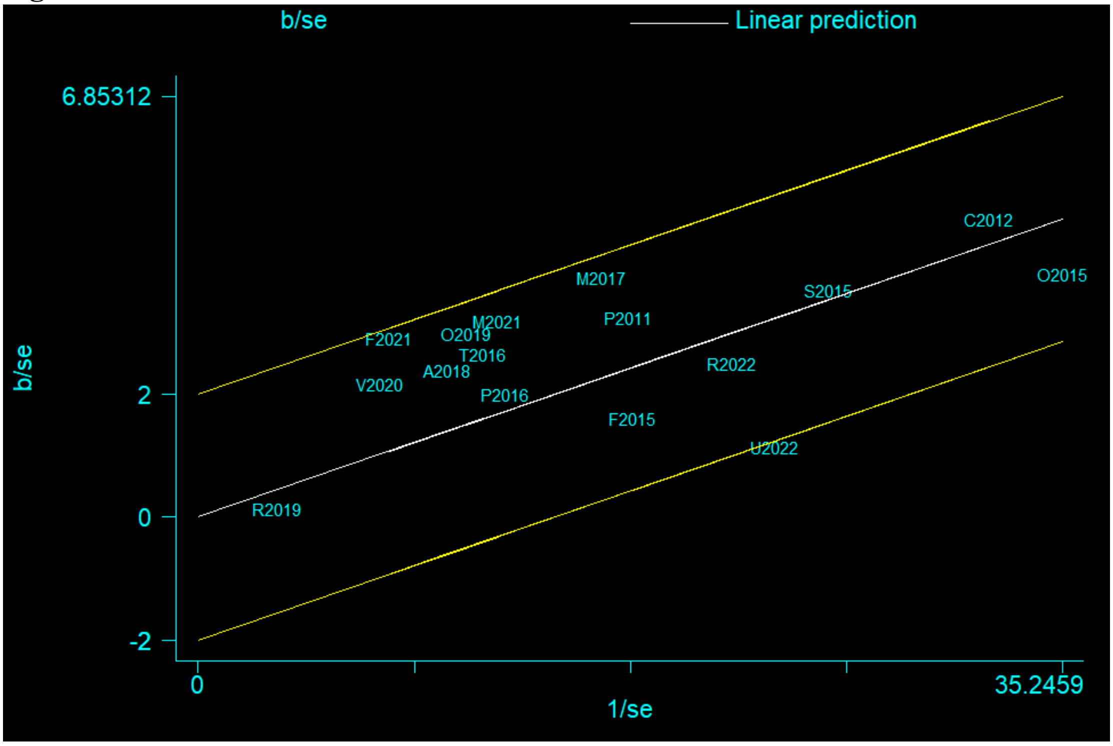

Figure S14. The Galbraith plot of complete response rate of relapsed/refractory peripheral T-cell lymphoma.

Figure S15.

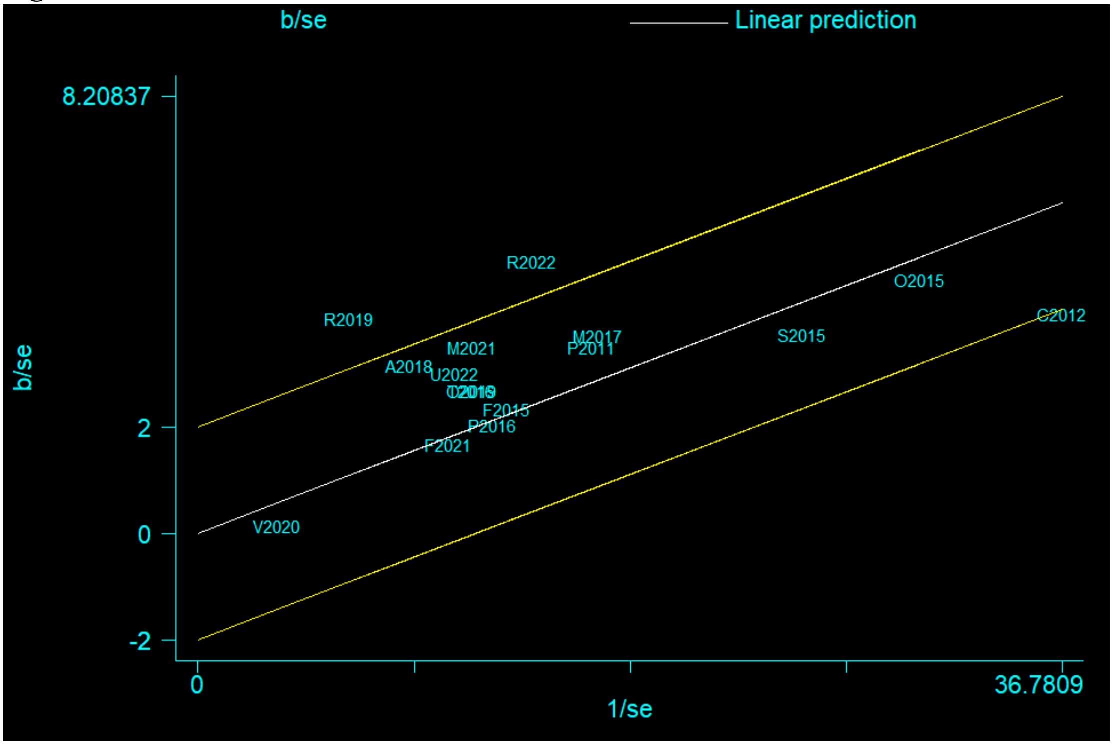

Figure S15. The Galbraith plot of partial response rate of relapsed/refractory peripheral T-cell lymphoma.

Figure S16.

Study or Subgroup	Risk Difference	SE	Weight	Risk Difference IV, Random, 95% CI
**Belinostat-based combination therapy**				
Johnston 2021	0.857143	0.07636	14.3%	0.86 [0.71, 1.01]
**Subtotal (95% CI)**			**14.3%**	**0.86 [0.71, 1.01]**
Heterogeneity: Not applicable				
Test for overall effect: Z = 11.23 (P < 0.00001)				
**Chidamide-based combination therapy**				
Gui 2021	0.892857	0.058451	16.5%	0.89 [0.78, 1.01]
Wang 2022	0.764706	0.05144	17.4%	0.76 [0.66, 0.87]
Zhang 2021	0.585938	0.043537	18.3%	0.59 [0.50, 0.67]
**Subtotal (95% CI)**			**52.2%**	**0.74 [0.57, 0.92]**
Heterogeneity: Tau² = 0.02; Chi² = 18.97, df = 2 (P < 0.0001); I² = 89%				
Test for overall effect: Z = 8.23 (P < 0.00001)				
**Romidepsin-based combination therapy**				
Bachy 2022	0.630332	0.033232	19.4%	0.63 [0.57, 0.70]
Dupuis 2015	0.685714	0.078469	14.1%	0.69 [0.53, 0.84]
**Subtotal (95% CI)**			**33.5%**	**0.64 [0.58, 0.70]**
Heterogeneity: Tau² = 0.00; Chi² = 0.42, df = 1 (P = 0.52); I² = 0%				
Test for overall effect: Z = 20.87 (P < 0.00001)				
**Total (95% CI)**			**100.0%**	**0.73 [0.63, 0.83]**
Heterogeneity: Tau² = 0.01; Chi² = 27.87, df = 5 (P < 0.0001); I² = 82%				
Test for overall effect: Z = 14.34 (P < 0.00001)				
Test for subgroup differences: Chi²=7.69, df=2 (P=0.02). I²=74.0%				

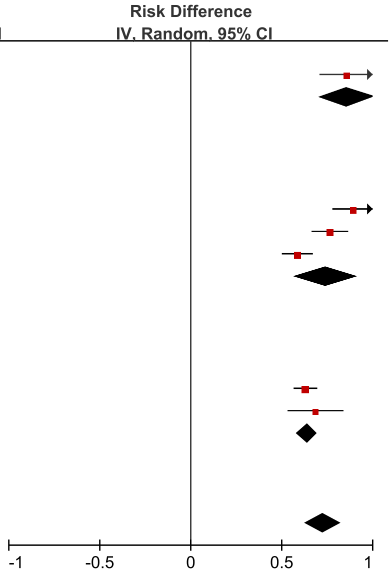

Figure S16. The sensitivity analysis by omitting one low-quality study in untreated peripheral T-cell lymphoma (overall response rate).

Figure S17.

Study or Subgroup	Risk Difference	SE	Weight	Risk Difference IV, Fixed, 95% CI
**Combination therapy**				
Pellegrini 2016	0.3	0.10247	3.5%	0.30 [0.10, 0.50]
Reiman 2019	0.6	0.154919	1.5%	0.60 [0.30, 0.90]
Tan 2015	0.434783	0.103367	3.5%	0.43 [0.23, 0.64]
Vu 2020	0.272727	0.134282	2.1%	0.27 [0.01, 0.54]
**Subtotal (95% CI)**			**10.6%**	**0.38 [0.27, 0.50]**
Heterogeneity: $Chi^2 = 3.54$, df = 3 (P = 0.32); $I^2 = 15\%$				
Test for overall effect: Z = 6.47 (P < 0.00001)				
**Monotherapy**				
Coiffier 2012	0.253846	0.038171	25.4%	0.25 [0.18, 0.33]
Foss 2015	0.25	0.088388	4.7%	0.25 [0.08, 0.42]
Maruyama 2017	0.4375	0.071603	7.2%	0.44 [0.30, 0.58]
O'Connor 2015	0.258333	0.039958	23.2%	0.26 [0.18, 0.34]
O'Connor 2019	0.478261	0.104159	3.4%	0.48 [0.27, 0.68]
Rai 2022	0.456522	0.073442	6.9%	0.46 [0.31, 0.60]
Shi 2015	0.278481	0.050432	14.6%	0.28 [0.18, 0.38]
Utsunomiya 2022	0.304348	0.095944	4.0%	0.30 [0.12, 0.49]
**Subtotal (95% CI)**			**89.4%**	**0.30 [0.26, 0.34]**
Heterogeneity: $Chi^2 = 14.21$, df = 7 (P = 0.05); $I^2 = 51\%$				
Test for overall effect: Z = 14.75 (P < 0.00001)				
**Total (95% CI)**			**100.0%**	**0.31 [0.27, 0.35]**
Heterogeneity: $Chi^2 = 19.50$, df = 11 (P = 0.05); $I^2 = 44\%$				
Test for overall effect: Z = 16.05 (P < 0.00001)				
Test for subgroup differences: $Chi^2=1.74$, df=1 (P=0.19). $I^2=42.5\%$				

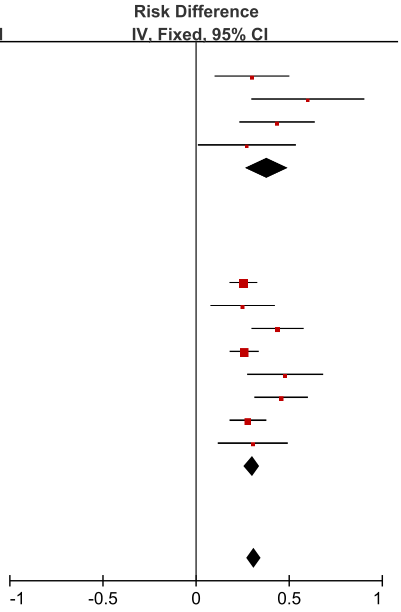

Figure S17. The sensitivity analysis by omitting four low-quality studies in relapsed/refractory peripheral T-cell lymphoma (overall response rate).
